# Molecular Phenotyping of Immune Cells from Young NOD Mice Reveals Abnormal Metabolic Pathways in the Early Induction Phase of Autoimmune Diabetes

**DOI:** 10.1371/journal.pone.0046941

**Published:** 2012-10-11

**Authors:** Jian Wu, Dorothy N. Kakoola, Nataliya I. Lenchik, Dominic M. Desiderio, Dana R. Marshall, Ivan C. Gerling

**Affiliations:** 1 Department of Medicine, University of Tennessee Health Science Center, Memphis, Tennessee, United States of America; 2 Research Service, Veterans Affairs Medical Center, Memphis, Tennessee, United States of America; 3 Department of Neurology, Department of Molecular Science, and the Charles B. Stout Neuroscience Mass Spectrometry Laboratory, University of Tennessee Health Science Center, Memphis, Tennessee, United States of America; 4 Department of Neurology, Xuan Wu Hospital, Capital Medical University, Beijing, China; 5 Department of Pathology, Anatomy and Cell Biology, School of Medicine, Meharry Medical College, Nashville, Tennessee, United States of America; Centro Cardiologico Monzino IRCCS, Italy

## Abstract

Islet leukocytic infiltration (insulitis) is first obvious at around 4 weeks of age in the NOD mouse – a model for human type 1 diabetes (T1D). The molecular events that lead to insulitis and initiate autoimmune diabetes are poorly understood. Since TID is caused by numerous genes, we hypothesized that multiple molecular pathways are altered and interact to initiate this disease. We evaluated the molecular phenotype (mRNA and protein expression) and molecular networks of *ex vivo* unfractionated spleen leukocytes from 2 and 4 week-old NOD mice in comparison to two control strains. Analysis of the global gene expression profiles and hierarchical clustering revealed that the majority (∼90%) of the differentially expressed genes in NOD mice were repressed. Furthermore, analysis using a modern suite of multiple bioinformatics approaches identified abnormal molecular pathways that can be divided broadly into 2 categories: metabolic pathways, which were predominant at 2 weeks, and immune response pathways, which were predominant at 4 weeks. Network analysis by Ingenuity pathway analysis identified key genes/molecules that may play a role in regulating these pathways. These included five that were common to both ages (TNF, HNF4A, IL15, Progesterone, and YWHAZ), and others that were unique to 2 weeks (e.g. MYC/MYCN, TGFB1, and IL2) and to 4 weeks (e.g. IFNG, beta-estradiol, p53, NFKB, AKT, PRKCA, IL12, and HLA-C). Based on the literature, genes that may play a role in regulating metabolic pathways at 2 weeks include Myc and HNF4A, and at 4 weeks, beta-estradiol, p53, Akt, HNF4A and AR. Our data suggest that abnormalities in regulation of metabolic pathways in the immune cells of young NOD mice lead to abnormalities in the immune response pathways and as such may play a role in the initiation of autoimmune diabetes. Thus, targeting metabolism may provide novel approaches to preventing and/or treating autoimmune diabetes.

## Introduction

The earliest signs of autoimmune pathology in the pancreas of the NOD mouse (a model for human type 1 diabetes, T1D) occur at approximately 4 weeks of age [1]. At this stage accumulation of leukocytes is observed around the pancreatic islets. This accumulation (known as insulitis) progressively intensifies and becomes increasingly more invasive eventually leading to massive destruction of the insulin producing beta cells with clinical diabetes occurring at 12 weeks of age or later [2], [3]. The molecular events leading to insulitis and that may influence initiation of the autoimmune pathology are poorly understood.

It is well established that genetic predisposition plays a major role in the etiology of TID. The strongest genetic determinant for T1D development is the MHC class II gene product, *H2^g7^* in NOD mice or HLA-DQ2 and –DQ8 in humans [2], [4], [5]. It is clear that MHC class II-restricted CD4 T-cells play an important role in inducing T1D [6]–[9]. In addition to the MHC locus, which is required for T1D development, over 20 non-MHC loci also contribute to the disease process [5], [10]–[13]. The molecular mechanisms by which most of these genes contribute to disease have not been elucidated. The focus in the field has been mainly to study candidate genes one by one. However, this approach has provided limited information on how the multiple genes interact to cause disease.

Since TID is caused by numerous genes, we hypothesized that multiple molecular pathways are altered and interact to initiate autoimmune pathology. To test this hypothesis, we conducted a detailed evaluation of the molecular phenotype (mRNA and protein expression) of untreated unfractionated spleen leukocytes from NOD mice in the pre-insulitis (early induction) phase of autoimmune diabetes. Applying a modern suite of multiple bioinformatics approaches, we identified abnormal molecular pathways that might play a role in the initiation of autoimmune pathology in NOD mice.

## Materials and Methods

### Ethics Statement

All the procedures involving animals were approved by the University of Tennessee (UT) Health Science Center and Veteran Affairs (VA) Medical Center Animal Care and Use Committee (Protocol Numbers: UT 1159/VA 00157). Breeder mice were purchased from the Jackson Laboratory. Animals were housed at the VA animal facility.

### Sample collection

The procedures for collection of spleen leukocytes, and extraction of RNA and protein samples have been previously described [14]. Spleen leukocytes were collected from female NOD mice and females of two control strains, NON, and C57BL/6, at 2 weeks and 4 weeks of age (n = 5 for each strain and each age group, 30 samples in total). NON is the original diabetes-resistant control strain closely related genetically to the NOD strain, whereas C57BL/6 is a more distantly related strain with some immunogenetic similarities to NOD. The isolated cells were not subjected to any post-collection treatment or culture prior to RNA/protein extraction.

### Transcriptome data collection

RNA expression was analyzed on Affymetrix expression arrays MOE430A and MOE430B as described previously [14]. Expression levels were evaluated using the Microarray Suite version 5.0 (MAS 5.0) using Affymetrix default analysis settings and global scaling as the normalization method (www.affymetrix.com). The trimmed mean target intensity of each array was arbitrarily set to 100. The two arrays were combined prior to statistical analysis to give a total of ∼45,000 probe sets. Microarray data has been submitted to the GEO repository (Accession number: GSE37450). The microarray results were validated by quantitative Real-time PCR (qRT-PCR) using Roche's Universal library probe system (www.roche-applied-science.com). First strand cDNA was synthesized from 1 µg of RNA using Transcriptor First Strand cDNA synthesis kit (Roche) according to the manufacturer's instructions. The target genes, primers and probes are listed in Table S1. Expression values were normalized using glyceraldehyde-3-phosphate dehydrogenase (Gapdh).

### Proteome data collection

Protein expression was analyzed by 2D PAGE as decribed previously [14]. The protein gels were scanned and analyzed as previously described [15], [16], except instead of using the PDQuest image analysis software to create the Match Set we used Nonlinear software (Progenesis). We identified a total of 382 protein spots as being expressed in at least one of the 30 gels.

### Statistical analysis

We imported and further analyzed the RNA and protein expression datasets in GeneSpring software (version 7.3.1, Silicon Genetics, Redwood, CA) as previously described [14]. The datasets consisted of 15 samples for each age, 2 weeks and 4 weeks. The 45,000 probe sets present on the Affymetrix expression arrays were filtered to identify 24,959 that had a present/marginal expression flag in at least one of the samples. The lists of filtered probe sets and the total protein spots were subjected to statistical and hierarchical clustering analysis. In order to define lists of age-specific differentially expressed genes between strains, we performed one-way ANOVA on the expression datasets by age. For the transcriptome datasets, we applied two cut-off levels, p<0.005, p<0.05, both with Benjamini-Hochberg multiple test correction. The proteome datasets were analyzed at one level p<0.05, with no multiple test correction (MTC). The impact of potential false positives that may arise from not applying a MTC on the final conclusions drawn from these data would be minimized by downstream data mining analyses and comparison with the transcriptome data. We conducted hierarchical clustering on the resulting lists to identify genes or spots that were differentially expressed only in the NOD mice compared to both control strains. We identified proteins in differentially expressed spots by mass spectrometry according to methods previously described [15].

### Data mining analyses

We subjected the lists of NOD differentially expressed mRNAs or proteins to data mining analyses using a suite of modern bioinformatics tools. The genes/proteins were grouped into functional classes to identify enriched gene ontology (GO) classes using WebGestalt Gene Set Analysis Toolkit (bioinfo.vanderbilt.edu/webgestalt) [17]. WebGestalt was also used for KEGG pathways analysis. Prior to uploading the lists into WebGestalt, the probe sets and proteins identifiers (ID) were converted to entrez-gene ID using the gene ID conversion tool available at the DAVID (Database for Annotation, Visualization and Integrated Discovery) server (david.abcc.ncifcrf.gov) [18], [19]. To identify molecular networks that are associated with the differentially expressed genes, we analyzed the gene lists using Ingenuity Pathway Analysis (IPA, www.ingenuity.com). The Affymetrix probe set IDs or GI numbers (for proteins) were uploaded into the server. Each identifier was mapped to its corresponding gene in the Ingenuity Pathways Knowledge Base (IPKB), a database compiled primarily from scientific literature. The mapped genes, called focus genes, were then algorithmically combined into molecular subnetworks together with other genes and/or endogenous chemicals from the IPKB (referred to as non-focus genes) based on information contained in the IPKB. The generated subnetworks are a graphical representation of the molecular relationships between genes/gene products and/or endogenous chemicals based on what is known about them from the literature. Herein, in the context of IPA, the term “gene” will be used for both genes/gene products as well as endogenous chemicals. Genes are represented as nodes of various shapes (shown in Figure S1) that represent the functional class of the gene product. The biological relationship between 2 nodes is represented as an edge (or line, also referred to in our study as a connection). Each connection is supported by at least one reference from the literature, a textbook, or from canonical information stored in the IPKB; and represents any known interaction between 2 genes (e.g. A induces B, C binds D, E inhibits F, and so on). Solid lines in the generated networks represent direct interactions (such as binding) while dashed lines represent indirect interactions (such as expression of gene A decreases expression of gene B). The Ingenuity package assesses the significance of each subnetwork with respect to random networks of the same size using the Fisher's exact test, and assigns a score based on the number of genes in the network and its size, the total number of genes analyzed, and the total number of genes in IPKB that could potentially be included in networks. We referred to the most highly scored preliminary networks as major networks. The biological processes and/or diseases most significantly associated to the genes in a particular network are identified using the IPA functional Analysis tool (Fisher's exact test). Because the Ingenuity server limits the number of genes per network to 35, bigger lists, like our transcriptome datasets, often create multiple networks. We think that this is an artificial division of the data which normally could be connected into one large network based on information available in the database. Therefore, we merged individual networks using the IPA “merge networks” tool. Due to the limitations of IPA (most important of which is the paucity or absence of information in the literature on particular genes) only a proportion of genes in the uploaded list may get incorporated into the generated network.

## Results

### Identification of differentially expressed mRNAs in spleen leukocytes from NOD mice at the preinsulitis stage of autoimmune diabetes

To identify differentially expressed mRNAs and proteins in spleen leukocytes from NOD mice at the preinsulitis stage, we analyzed transcriptome and proteome expression in 2 and 4 week-old NOD mice. Each dataset was analyzed by age. We compared gene expression in NOD mice to that in two control strains, NON and C57. We were interested in genes that were differentially expressed in NOD relative to both controls (hereinafter referred to as NOD differentially expressed genes or mRNAs or proteins). We judged that this strategy would increase the likelihood that the identified genes are linked to initiation of the autoimmune pathology seen in NOD mice.

**Figure 1:**
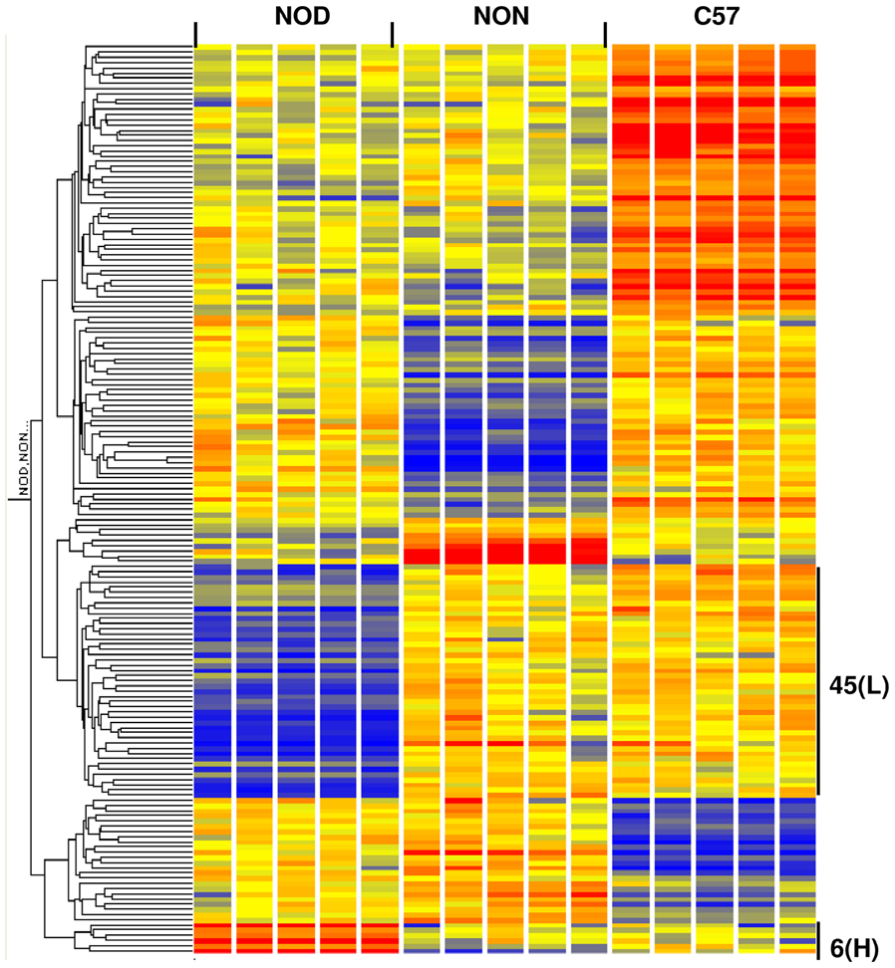
Hierarchical clustering of 175 genes with highly significant expression differences between strains at 2 weeks. The list was generated by one-way ANOVA of 24,959 probe sets (p<0.005, Benjamini-Hochberg multiple test correction). A total of 51 genes were differentially expressed in NOD mice relative to both control strains (NON and C57BL/6, C57): 45 were of lower expression (L) and 6 were of higher expression (H) in NOD mice. The color intensity of the rectangles representing each gene for each sample indicates the degree of increase (red) or decrease (blue) of the gene expression signal relative to the mean signal intensity (yellow).

**Figure 2:**
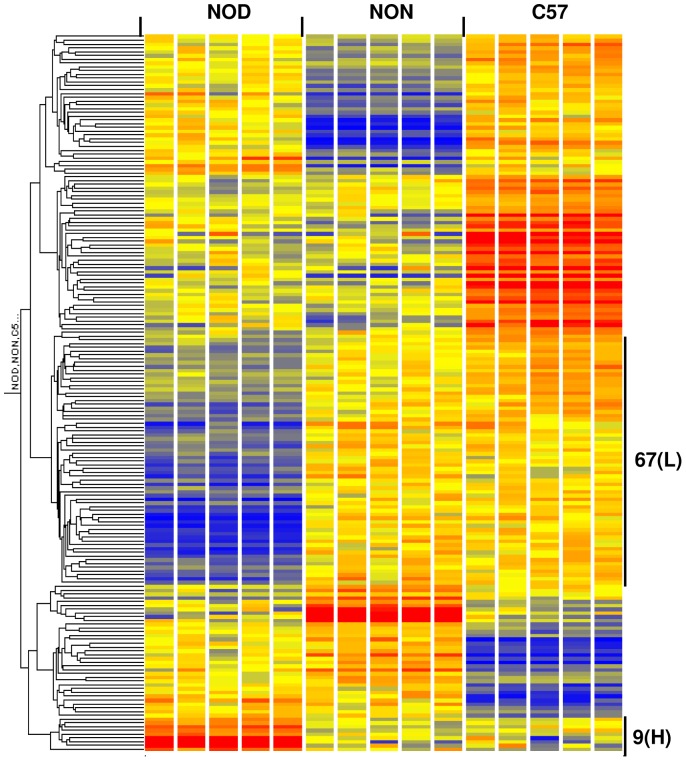
Hierarchical clustering of 189 genes with highly significant expression differences between strains at 4 weeks. The list was generated by one-way ANOVA of 24,959 probe sets (p<0.005, Benjamini-Hochberg multiple test correction). A total of 76 genes were differentially expressed in NOD mice relative to both control strains (NON and C57BL/6): 67 were of lower expression (L) and 9 were of higher expression (H) in NOD mice. The color intensity of the rectangles representing each gene for each sample indicates the degree of increase (red) or decrease (blue) of the gene expression signal relative to the mean signal intensity (yellow).

To identify differentially expressed mRNAs, we used expression microarrays and performed a one-way ANOVA on ∼25,000 filtered probe sets. We identified 175 and 189 probe sets (genes) with highly significant strain differences (p<0.005, Benjamini-Hochberg multiple test correction) at 2 and 4 weeks, respectively. We then subjected these two gene lists to hierarchical clustering as previously described [20]. The hierarchical clusters of the 2 week (Figure 1) and 4 week (Figure 2) datasets each revealed six distinct patterns of expression: NOD low, NOD high, NON low, NON high, C57 low, and C57 high. At 2 weeks, a total of 51 genes (marked by black bars in Figure 1, and listed in Table 1) were differentially expressed in NOD mice compared to both control strains: 45 (88%) were of lower expression while 6 (12%) were of higher expression in NOD. At 4 weeks, a total of 76 genes (marked by black bars in Figure 2 and listed in Table 2) were differentially expressed in NOD mice compared to both control strains: 67 (88%) were of lower expression while 9 (12%) were of higher expression in NOD mice. Twenty-five genes (88% of which was of lower expression in NOD mice) were common to both 2 and 4 week old mice. Microarray results were validated by qRT-PCR. Results confirming the microarray data for six genes (H60a, Trim5, Trim12, Ly6c1, Snx6 and Sp110) are shown in Figure S2. The major functional categories (as determined by us based on information obtained from literature searches) included immune response, apoptosis, ubiquitination, transporters, transcription and protein synthesis, and enzymes (including those involved in phosphorylation) at both ages. Several genes did not fall in one major group, thus were listed under “Other”. Probe sets of unknown identification/function are listed under “Unknown”. Of note, a number of these genes have been reported to be linked to T1D risk. For example, Ly6c1 and Trim 5 belong to a previously identified IRF7-driven expression network that is linked to TID risk [21]. Ly6c1, Trim 12 and Trim 30 are linked to a defect in T cell negative selection and have been previously identified as T1D candidate genes [22].

**Table 1:** Transcripts with highly significant expression differences in spleen leukocytes of 2 week old NOD mice compared to two control strains.

Probe Set ID	Gene Symbol	Fold Change	Adjusted p-value	Description
**Immune response**				
**1457769_at (H)**	**H60A**	**29.75**	**2.41e-5**	**histocompatibility 60a**
**1443858_at**	**Trim5**	**−20.14**	**0.000656**	**tripartite motif protein 5**
**1442338_at**	**Tnfrsf14**	**−8.68**	**0.00367**	**tumor necrosis factor receptor superfamily, member 14**
**1450355_a_at**	**Capg**	**−5.9**	**8.87e-5**	**capping protein (actin filament), gelsolin-like**
**1421571_a_at**	**Ly6c1**	**−5.15**	**0.00029**	**lymphocyte antigen 6 complex, locus C1**
1437937_at (H)	Ccbp2	2.77	0.000834	Chemokine binding protein 2
1449991_at (H)	Cd244	2.74	0.00099	CD244 molecule, natural killer cell receptor 2B4
**Apoptosis**				
**1423747_a_at**	**Pdk1**	**−5.14**	**6.47e-5**	**pyruvate dehydrogenase kinase, isoenzyme 1**
1438454_at	Pten	−4.22	0.000953	Phosphatase and tensin homolog
**Ubiquitination**				
**1437432_a_at**	**Trim12**	**−26.06**	**3.39e-5**	**tripartite motif protein 12**
**1437007_x_at**	**Usp39**	**−7.80**	**6.47e-5**	**ubiquitin specific protease 39**
1446608_at	Cbl	−5.98	0.00487	Casitas B-lineage lymphoma
1428592_s_at	Usp38	−2.38	0.000396	ubiquitin specific protease 38
**Transporters**				
**1451602_at**	**Snx6**	**−17.31**	**0.000834**	**sorting nexin 6**
1417600_at	Slc15a2	−6.88	0.00179	solute carrier family 15 (H+/peptide transporter), member 2
**1416629_at**	**Slc1a5**	**−4.53**	**0.00428**	**solute carrier family 1, member 5**
1418473_at	Cutc	−1.79	0.00391	cutC copper transporter homolog (E.coli)
**Transcription**				
1439780_at	Rpl7l1	−6.12	0.000895	ribosomal protein L7-like 1
**1449648_s_at**	**Rpo1-1**	**−3.86**	**0.00109**	**RNA polymerase 1-1 (Ingenuity symbol: POLR1C)**
1424170_at	Phf5a	−2.16	0.000608	PHD finger protein 5A
**Protein biosynthesis**				
**1454714_x_at**	**Phgdh**	**−10.28**	**3.11e-5**	**phosphoglycerate dehydrogenase**
1448472_at	Vars	−4.14	0.000382	valyl-tRNA synthetase 2
**1417762_a_at**	**Rpl8**	**−2.13**	**0.00348**	**ribosomal protein L8**
1455887_at	Alg8	−1.17	0.000555	Asparagine-linked gylcosylation 8 homolog
**Phosphorylation**				
1446130_at	Pctk2	−7.98	0.00453	PCTAIRE-motif protein kinase 2
**1416770_at**	**Stk25**	**−2.98**	**0.000365**	**serine/threonine kinase 25 (yeast)**
1448450_at	Ak2	−1.60	0.00206	adenylate kinase 2
**Enzymes**				
1421802_at	Ear1	−103.15	0.00138	eosinophil-associated, ribonuclease A family, member 1
**1416494_at**	**Ndufs5**	**−6.89**	**0.000102**	**NADH dehydrogenase (ubiquinone) Fe-S protein 5**
1427441_a_at	Suclg2	−2.64	0.000354	succinate-Coenzyme A ligase, GDP-forming, beta subunit
1451277_at	Zadh2	−1.50	0.00236	zinc binding alcohol dehydrogenase, domain containing 2
**Other**				
**1417461_at (H)**	**Cap1**	**25.17**	**3.96e-5**	**CAP, adenylate cyclase-associated protein 1 (yeast)**
**1456635_at**	**Sp110**	**−9.89**	**3.07e-5**	**Sp110 nuclear body protein**
**1416479_a_at**	**Tmem14c**	**−9.30**	**2.41e-5**	**transmembrane protein 14C**
1435553_at	Pdzd2	−9.25	0.000938	PDZ domain containing 2
1416503_at	Lxn	−3.32	0.00231	Latexin
**1434441_at**	**C9orf21**	**−3.23**	**7.15e-5**	**chromosome 9 open reading frame 21(peroxiredoxin (PRX)-like 2 family)**
**1436061_at**	**Chaf1a**	**−3.03**	**0.00139**	**chromatin assembly factor 1, subunit A (p150)**
**1435745_at**	**5031439G07Rik**	**−2.94**	**0.00164**	**chromosome 22 open reading frame 9 (C22ORF9)**
**1435660_at (H)**	**LOC434484**	**2.79**	**0.000271**	**similar to SP140 nuclear body protein; nuclear body protein Sp140**
1418072_at	Hist1h2bi	−2.77	0.000993	histone cluster 1, H2bi
1459827_x_at	Hps1	−2.40	0.00083	Hermansky-Pudlak syndrome 1 homolog (human)
1436836_x_at	Cnn3	−2.00	7.15e-5	calponin 3, acidic
**Unknown**				
1426936_at	LOC215866	−15.28	0.000834	Mus musculus, cDNA clone IMAGE:3493956, mRNA
**1417822_at**	**D17H6S56E-5**	**−7.76**	**6.43e-7**	**DNA segment, Chr 17, human D6S56E 5**
**1440214_at**	**A630001G21Rik**	**−4.04**	**0.00083**	**RIKEN cDNA A630001G21 gene**

Statistical analysis of mRNA expression data by 1-way-ANOVA (Benjamini-Hochberg, p<0.005) followed by hierarchical clustering identified 51 probe sets (representing 47 different genes) that were differentially expressed in spleen leukocytes of 2 week old NOD mice compared to two control strains (NON and C57BL/6); H, indicates 5 genes (∼11%) with significantly higher expression in NOD relative to controls; the rest, 42 (∼89%) had significantly lower expression in NOD. Fold change (FC) was calculated by ratio of means of expression in NOD mice versus controls; FC of genes of lower expression in NOD are indicated by negative values. We assigned the genes to selected functional categories based on information obtained by searches of public databases. Genes highlighted with **bold font** were differentially expressed at both 2 and 4 weeks.

**Table 2:** Transcripts with highly significant expression differences in spleen leukocytes of 4 week old NOD mice compared to two control strains.

Probe Set ID	Gene Symbol	Fold Change	Adjusted p-value	Description
**Immune response**				
**1457769_at (H)**	**H60A**	**26.55**	**7.49e-5**	**histocompatibility 60a**
**1443858_at**	**Trim5**	**−17.29**	**0.00179**	**tripartite motif protein 5**
**1442338_at**	**Tnfrsf14**	**−6.78**	**0.000529**	**tumor necrosis factor receptor superfamily, member 14**
**1421571_a_at**	**Ly6c1**	**−4.61**	**1.76e-5**	**lymphocyte antigen 6 complex, locus C1**
1427799_x_at (H)	Igk	4.46	0.000109	immunoglobulin kappa chain variable 21 (V21)-4
**1450355_a_at**	**Capg**	**−3.95**	**3.64e-6**	**capping protein (actin filament), gelsolin-like**
1451965_at	Igk	−3.65	0.000255	Mouse mRNA for immunoglobulin kappa variable region
1424948_x_at (H)	H2-D1	2.89	0.00039	major histocompatibility complex, class I, C
1422188_s_at	Tcrg-V4	−2.81	0.00156	T-cell receptor gamma, variable 4
1426112_a_at	Cd72	−2.55	0.00206	CD72 antigen
1425477_x_at (H)	H2-Ab1	2.40	0.00402	major histocompatibility complex, class II, DQ beta 2
1425436_x_at	Klra7	−2.30	0.000858	killer cell lectin-like receptor, subfamily A, member 7
1448706_at	Ttrap	−2.08	0.00344	Traf and Tnf receptor associated protein
1442219_at	Ms4a4b	−2.07	0.00206	membrane-spanning 4-domains, subfamily A, member 4B
1456426_at	Clec2i	−1.93	0.0029	C-type lectin domain family 2, member i
1460204_at	Tec	−1.89	0.00292	cytoplasmic tyrosine kinase, Dscr28C related (Drosophila)
1419481_at	Sell	−1.88	0.00402	selectin, lymphocyte
1460431_at	Gcnt1	−1.80	0.00234	glucosaminyl (N-acetyl) transferase 1, core 2
1448862_at	Icam2	−1.66	0.0031	intercellular adhesion molecule 2
**Apoptosis**				
1431708_a_at	Tia1	−12.96	2.25e-5	TIA1 cytotoxic granule-associated RNA binding protein 1
**1423747_a_at**	**Pdk1**	**−6.65**	**1.99e-5**	**pyruvate dehydrogenase kinase, isoenzyme 1**
1455287_at	Cdk6	−2.74	0.00276	Cyclin-dependent kinase 6
1437393_at	Prkca	−2.28	0.00282	protein kinase C, alpha
1434312_at	Arf6	−1.98	0.00342	ADP-ribosylation factor 6
1419706_a_at	Akap12	−1.88	0.00319	A kinase (PRKA) anchor protein (gravin) 12
**Ubiquitination**				
**1437432_a_at**	**Trim12**	**−51.45**	**6.17e-6**	**tripartite motif protein 12**
**1437007_x_at**	**Usp39**	**−5.28**	**0.00123**	**ubiquitin specific protease 39**
1424857_a_at	Trim34	−2.64	6.22e-5	tripartite motif protein 34
**Transporters**				
**1451602_at**	**Snx6**	**−8.74**	**0.00377**	**sorting nexin 6**
**1416629_at**	**Slc1a5**	**−2.97**	**0.000477**	**solute carrier family 1, member 5**
1426775_s_at (H)	Scamp1	2.33	0.00269	secretory carrier membrane protein 1
1433898_at	Slc25a30	−1.93	0.00261	solute carrier family 25, member 30
1460224_at	Snx2	−1.63	0.00427	sorting nexin 2
**Transcription**				
1440845_at	Dmtf1	−12.13	3.64e-6	cyclin D binding myb-like transcription factor 1
1441823_at	Ria17	−7.04	0.000744	retinoic acid induced 17/Zinc finger, MIZ-type containing 1
**1449648_s_at**	**Rpo1-1**	**−3.19**	**0.00241**	**RNA polymerase 1-1**
1417961_a_at	Trim30	−2.99	0.00123	tripartite motif protein 30
1428662_a_at	Hopx	−2.24	0.00354	homeobox only domain, MGI:1916782
1433599_at	Baz1a	−1.96	0.00343	bromodomain adjacent to zinc finger domain 1A
**Protein biosynthesis**				
**1454714_x_at**	**Phgdh**	**−5.33**	**3.14e-5**	**phosphoglycerate dehydrogenase**
**1417762_a_at**	**Rpl8**	**−2.01**	**0.00015**	**ribosomal protein L8**
1420994_at	B3gnt5	−1.89	0.00292	UDP-GlcNAc:betaGal beta-1,3-N-acetylglucosaminyltransferase 5
**Phosphorylation**				
1451253_at	Pxk	−2.99	0.00142	PX domain containing serine/threonine kinase
**1416770_at**	**Stk25**	**−2.35**	**0.00214**	**serine/threonine kinase 25 (yeast)**
**Enzymes**				
**1416494_at**	**Ndufs5**	**−7.24**	**1.33e-5**	**NADH dehydrogenase (ubiquinone) Fe-S protein 5**
1448942_at (H)	Gng11	3.64	0.00247	guanine nucleotide binding protein (G protein), gamma 11
1447458_at	St3gal4	−2.92	0.00214	ST3 beta-galactoside alpha-2,3-sialyltransferase 4
1453109_at	Arsk	−2.87	0.00113	Arylsulfatase K
1455734_at	Crbn	−2.41	0.00214	cereblon
1436494_x_at	Trmt1	−2.23	0.00478	Trm1 tRNA methyltransferase 1homolog (yeast)
1417316_at	Them2	−1.81	0.00214	thioesterase superfamily member 2
1424043_at	Ppil4	−1.58	0.00223	peptidylprolyl isomerase (cyclophilin)-like 4
**Other – partial list; other genes are listed below in the legend.**				
**1417461_at (H)**	**Cap1**	**29.41**	**0.00017**	**CAP, adenylate cyclase-associated protein 1 (yeast)**
**1456635_at**	**Sp110**	**−13.82**	**2.10e-5**	**Sp110 nuclear body protein**
**1416479_a_at**	**Tmem14c**	**−7.51**	**6.15e-5**	**Transmembrane protein 14C**
**1436061_at**	**Chaf1a**	**−7.05**	**0.00185**	**chromatin assembly factor 1, subunit A (p150)**

Statistical analysis of mRNA expression data by 1-way-ANOVA (Benjamini-Hochberg, p<0.005) followed by hierarchical clustering identified 76 probe sets (representing 72 different genes) that were differentially expressed in spleen leukocytes of 4 week old NOD mice compared to two control strains (NON and C57BL/6); H, indicates 8 genes (∼11%) with significantly higher expression in NOD relative to controls; the rest, 68 (∼89%) had significantly lower expression in NOD. Fold change (FC) was calculated by ratio of means of expression in NOD mice versus controls; FC of genes of lower expression in NOD are indicated by negative values. We assigned the genes to selected functional categories based on information obtained by searches of public databases. Other genes under “Other” include: Fcrla, Otu1, Ankrd16, Treml2, Plekha2, Fam3c, 0610039P13Rik, Fam65b, Pyhin1, **5031439G07Rik, C9orf21, LOC434484 (H)**; Unknown genes include: **D17H6S56E-5, A630001G21Rik**, A530030E21Rik. Genes highlighted with **bold font** were differentially expressed at both 2 and 4 weeks.

To identify genes likely associated directly with initiation of insulitis, we compared the trascriptomes of 2 week old NOD mice with those of 4 week old mice, with reference to corresponding comparative analyses of the control strains. Statistical analysis of the mRNA expression datasets by unpaired t-test (p<0.001, no MTC) followed by Venn diagram analysis identified 66 probe sets (representing 65 different genes, Table 3), 122 probesets (representing 120 genes, Table S2) and 105 probesets (representing 104 genes; Table S3) that were uniquely differentially expressed in spleen leukocytes of NOD, NON and C57BL/6 mice, respectively. Expression of ∼62% [40] of the NOD-specific age differences was upregulated at 4 weeks compared to expression at 2 weeks of age (Table 3), in contrast to 45% and ∼26% in NON and C56BL/6 mice, respectively. Unlike the gene lists of the control strains, the list of NOD mice consisted of several molecules involved in immune activation (e.g. Plcd4, Mapk4, Ifit3, Pdcd1lg2, Ifih1, IL17rb).

**Table 3:** Transcripts significantly differentially expressed in spleen leukocytes between 2 weeks and 4 weeks of age in NOD mice but not in two control strains.

Probe Set ID	Gene Symbol	Fold Change	Regulation at 4 wks	p-value	Description
1459452_at	Cml2	27.32	up	0.000959	camello-like 2
1418086_at	Ppp1r14a	15.68	up	0.001	protein phosphatase 1, regulatory (inhibitor) subunit 14A
1424631_a_at	Ighg	11.77	up	7.21e-5	Immunoglobulin heavy chain (gamma polypeptide)
1435987_x_at	1110059G02Rik	8.3	up	0.000645	RIKEN cDNA 1110059G02 gene
1440771_at	Zkscan1	−7.59	down	0	zinc finger with KRAB and SCAN domains 1
1431640_at	4933431J24Rik	7.37	up	0.00056	RIKEN cDNA 4933431J24 gene
1432928_at	Plcd4	6.93	up	5.25e-6	phospholipase C, delta 4
1425385_a_at	Ighm	6.9	up	0.000779	immunoglobulin heavy constant mu
1437986_x_at	Fuk	6.63	up	9.22e-5	fucokinase
1444140_at	Pum1	−6.43	down	0.000796	pumilio 1 (PUF family RNA-binding protein)
1452358_at	Rai2	5.67	up	7.14e-5	retinoic acid induced 2
1426174_s_at	Ighg	5.45	up	0.000444	Immunoglobulin heavy chain (gamma polypeptide)
1423243_at	Mpp1	−4.79	down	0.000209	membrane protein, palmitoylated 1, 55kDa
1432782_at	4933435C09Rik	4.63	up	4.78e-5	RIKEN cDNA 4933435C09 gene
1441524_at	Tec	4.53	up	0.001085	cytoplasmic tyrosine kinase, Dscr28C related (Drosophila)
1439855_at	Tmtc1	−4.48	down	0.0003	transmembrane and tetratricopeptide repeat containing 1
1426547_at	Gc	4.21	up	1.41e-5	group-specific component (vitamin D binding protein)
1435367_at	Mapk4	−4.08	down	0.000806	mitogen-activated protein kinase 4
1453957_a_at	Igf2bp3	−4.03	down	0.00029	insulin-like growth factor 2 mRNA binding protein 3
1441710_at	4930479D17Rik	−3.57	down	0.001082	RIKEN cDNA 4930479D17 gene
1447934_at	C12orf5	−3.55	down	0.000111	chromosome 12 open reading frame 5
1451492_at	Sla2	3.53	up	7.55e-5	Src-like-adaptor 2
1441955_s_at	Paip1	3.30	up	0.000908	poly(A) binding protein interacting protein 1
1444416_at	CenpA	−3.23	down	0.000564	centromere protein A
1432162_s_at	Ptar1	3.10	up	0.000509	protein prenyltransferase alpha subunit repeat containing 1
1453760_at	Mier1	−3.01	down	0.000385	mesoderm induction early response 1 homolog (Xenopus laevis)
1432530_s_at	Boll	2.97	up	0.000906	bol, boule-like (Drosophila)
1430717_at	Fam83e	2.84	up	0.000592	family with sequence similarity 83, member E
1449306_at	Hsf2	2.67	up	0.000948	heat shock transcription factor 2
1449306_at	Rps4y2	−2.62	down	0.000292	ribosomal protein S4, Y-linked 2
1434405_at	Fnip1	−2.46	down	0.000305	folliculin interacting protein 1
1459227_at	953001807Rik	2.40	up	0.000204	RIKEN cDNA 9530018I07 gene
1429754_a_at	1700110I01Rik	2.40	up	0.000381	predicted gene 5957
1426041_a_at	Fgd4	−2.34	down	0.000524	FYVE, RhoGEF and PH domain containing 4
1442315_at	Foxd2	2.28	up	0.000931	forkhead box D2
1446698_at	Lpp	−2.27	down	0.000523	LIM domain containing preferred translocation partner in lipoma
1449153_at	Mmp12	2.22	up	0.000549	matrix metallopeptidase 12 (macrophage elastase)
1437230_at	Kcna1	−2.22	down	0.001029	potassium voltage-gated channel, shaker-related subfamily, member 1 (episodic ataxia with myokymia)
1426218_at	Glcci1	−2.19	down	0.000131	glucocorticoid induced transcript 1
1424698_s_at	Gca	−2.13	down	0.001086	grancalcin, EF-hand calcium binding protein
1420731_a_at	Csrp2	−2.05	down	9.09e-5	cysteine and glycine-rich protein 2
1427471_at	Fbxl3	−2.02	down	0.000372	F-box and leucine-rich repeat protein 3
1427718_a_at	Mdm2	−1.97	down	0.001089	Mdm2, p53 E3 ubiquitin protein ligase homolog (mouse)
1420330_at	Clec4e	1.94	up	0.000866	C-type lectin domain family 4, member E
1435856_x_at	Smarcb1	−1.89	down	0.000589	SWI/SNF related, matrix associated, actin dependent regulator of chromatin, subfamily b, member 1
1416107_at	Hmp19	1.83	up	0.001088	HMP19 protein, neuron specific gene family member 2
1425294_at	Slamf8	1.83	up	0.000142	SLAM family member 8
1443884_at	Thada	1.80	up	0.00066	thyroid adenoma associated
1449025_at	Ifit3	1.73	up	0.000955	interferon-induced protein with tetratricopeptide repeats 3
1419634_a_at	Ghrh	1.73	up	0.000456	growth hormone releasing hormone
1450290_at	Pdcd1lg2	1.67	up	0.000798	programmed cell death 1 ligand 2
1426276_at	Ifih1	1.64	up	0.000163	interferon induced with helicase C domain 1
1454799_at	AgpaT9	−1.64	down	0.000332	1-acylglycerol-3-phosphate O-acyltransferase 9
1460651_at	Lat	1.61	up	0.000971	linker for activation of T cells
1416087_at	Ap1s1	−1.50	down	0.000297	adaptor-related protein complex 1, sigma 1 subunit
Genes with fold change of <1.5 and their regulation at 4 weeks are listed in the legend.					

Statistical analysis of mRNA expression data by unpaired t-Test (p<0.001) followed by Venn diagram analysis identified 66 probe sets (representing 65 different genes) that were uniquely differentially expressed in spleen leukocytes of NOD mice between 2 and 4 weeks of age in comparison to two control strains, NON and C57BL/6. Fold change (FC) was calculated by ratio of means of expression at 2 weeks versus 4 weeks; FC of genes of lower expression at 4 weeks (compared to 2 weeks) are indicated by negative values. Genes with FC<1.5 (and their FC values) included: Syt11 (1.47), Snap47 (1.46), Cklf (−1.45), Cd274 (1.43), Heatr7a (1.42), Fam76b (−1.41), Npsr1 (1.39), Nrn1 (1.38), Eef2k (1.24), Tomm34 (1.21), and IL17rb (1.2). Expression of approximately 62% (40) of the genes was upregulated at 4 weeks compared to expression at 2 weeks of age.

### Identification of differentially expressed proteins in spleen leukocytes from NOD mice at the preinsulitis stage of autoimmune diabetes

To identify differentially expressed proteins, we analyzed protein extracts that were simultaneously obtained from the same samples that generated the RNA as described previously [14]. Supplemental figure S3 shows an example of the 2D-gels that were produced by the protein extracts. We conducted a one-way ANOVA on a total of 382 spots to identify proteins with statistically significant (p<0.05) expression differences between strains. We found that 19 and 26 spots had strain differences at 2 and 4 weeks, respectively, of which 11 and 8, respectively, were differentially expressed in NOD compared to both control strains. Of the proteins differentially expressed in NOD only, we identified 9 at two weeks (6 lower, 3 higher in NOD) and 7 at 4 weeks (4 lower, 3 higher) (Table 4). In some cases, we identified two proteins from a single spot (423, 441, 523), likely due to proximity in their respective molecular weights. In other cases, a single protein was identified from two different spots (Lmnb1 and Lcp1 from 423 and 532, and Hspa8 from 471 and 601), likely due to post-translational modifications of the protein. Although, no proteins were common to the proteome datasets of 2 and 4 week old mice, our functional categorization showed that apoptosis (the major functional category represented) was common to both age groups.

**Table 4:** Proteins with significant expression differences in spleen leukocytes of 2 week and 4 week old NOD mice compared to two control strains and their expression differences at RNA level.

Spot ID	Fold Change (FC)	Proteins identified	p-value at RNA level (FC)	Description
**2 week**				
149 (H)	3.08	Clip1	NS	Cap-Gly domain containing linker protein 1
190	−2.39	Hsp90ab1	0.02 (−1.26)	Heat shock protein1, beta
532	−1.82	Lmnb1 and Lcp1	NS	Lamin B1 and Lymphocyte cytosolic protein 1, respectively
441	−1.80	Tuba1 and Vim	NS	Tubulin alpha 1 and Vimentin, respectively
24 (H)	1.78	Hspa9	0.03 (−1.22)	Heat shock protein 9A
423	−1.74	Lmnb1 and Lcp1	NS	Lamin B1 and Lymphocyte cytosolic protein 1, respectively
471	−1.65	Hspa8	NS	Heat shock protein 8
601	−1.50	Hspa8	NS	Heat shock protein 8
618 (H)	1.33	Gopc	NS	Golgi associated PDZ and coiled-coil motif containing
45 (H)	1.74	unidentified	unknown	not available
702 (H)	1.36	unidentified	unknown	not available
**4 week**				
74 (H)	6.61	Arhgdib	NS	Rho, GDP dissociation inhibitor (GDI) beta
72 (H)	6.44	S100a5	0.03 (1.78)	S100 calcium binding protein A5
132	−3.45	Ctnna1	NS	Catenin alpha 1
295 (H)	2.11	Hspd1	NS	Heat shock protein 1(chaperonin)
430	−2.00	Ppp5c	NS	Protein phosphatase 5, catalytic subunit
390	−1.91	Serpinb1a	0.006 (−1.37)	Serine (or cysteine) proteinase inhibitor, clade B, member 1a
225	−1.88	Wdr1	NS	WD repeat domain 1
485	−1.83	unidentified	NS	not available

Statistical analysis of protein expression data by 1-way-ANOVA (p<0.05) followed by hierarchical clustering identified 11 and 8 spots that were differentially expressed in spleen leukocytes of 2 week and 4 week old NOD mice, respectively, compared to two control strains (NON and C57BL/6); H, indicates spots that were of higher expression in NOD, all other spots were of lower expression in NOD mice. We identified proteins in 9 and 7 of the spots at 2 and 4 weeks, respectively. We also analyzed expression differences of the identified proteins at the RNA level using the microarray (mRNA) dataset; NS, indicates no significance in gene expression between NOD and control strains. Fold change (FC) was calculated by ratio of means of expression in NOD mice versus controls; FC of spots/genes of lower expression in NOD are indicated by negative values.

### Integration of the transcriptome and proteome data

Inspection of the lists of proteins or transcripts that were differentially expressed in NOD relative to control strains revealed no overlap between them. While expression of a gene at the mRNA and protein level does not always correlate, other factors may be at play in our study, including: a) protein spots detected on 2D gels do not represent the total expression of the gene at the protein level but often just one of many post-translationally modified form of the protein; b) the protein analysis only included proteins within the pI range of 4–7; c) the percentage of the total proteome covered was much smaller than that the percentage of the transcriptome; and d) different statistical thresholds were applied to the two datasets (Benjamini-Hochberg, p<0.005 vs. no MTC, p<0.05). To this end, we analyzed gene expression at the RNA level of the 16 proteins identified (at 2 and 4 weeks combined) using the statistical cut-off level that was applied to the proteome data set. We found that 4 (Hsp90ab1, Hspa9, S100a5 and Serpinb1a) were also differentially expressed at the transcript level (Table 4). However, the majority were significantly differentially expressed only at the protein level. Thus, investigation at the proteome level allowed us to capture additional genes whose expression may be altered in NOD mice spleen leukocytes. Overall, the data indicate that the vast majority of genes (88%) that are abnormally expressed in NOD spleen leukocytes in comparison to control strains are repressed and that regulation of abnormal expression may occur at either the RNA or protein level.

### Gene ontology (GO) enrichment analysis

To further understand the biological processes associated with abnormalities in the NOD mouse, we performed Gene ontology (GO) analysis of the NOD-specific strain differences using WebGestalt. Because GO analysis is better suited for larger gene lists, we used a slightly less stringent analysis for the transcriptome datasets to obtain expanded lists. Such bigger lists minimize the number of false negatives and increase the efficiency of GO classification. Thus, one-way ANOVA at cut-off p<0.05 with Benjamini-Hochberg followed by hierarchical clustering identified 187 (87% were of lower expression in NOD) and 327 (91% were of lower expression in NOD) NOD differentially expressed genes at 2 and 4 weeks, respectively. All the genes, including FC and adjusted p-values, are listed in Tables S4 (2 weeks) and S5 (4 weeks). Functional clustering of these genes revealed that the top ranked major biological process was metabolism (Table 5). The major unique biological process terms at 2 weeks were biosynthesis, amine metabolism and RNA processing, while those at 4 weeks were regulation of biological processes, immune system process (further categorized into defense response, and antigen processing and presentation), and apoptosis. Under the molecular function GO (Table 6), the common (i.e. enriched in both the 2 and 4 week gene lists) top ranked term was transferase activity. Catalytic activity was uniquely enriched at 2 weeks, while binding (accounting for more than half of the genes) was uniquely enriched at 4 weeks. Kinase activity was also a significant category but only at 4 weeks. The most salient features under the cellular component category (Table 6) were significant enrichment for mitochondrion at 2 weeks, and nucleus and external side of plasma membrane (including MHC protein complex) at 4 weeks. Gene Ontology analysis of the NOD differentially expressed proteins revealed involvement of the cytoskeleton, protein binding and response to stress.

**Table 5:** Enriched biological process Gene ontology (GO) categories for the lists of differentially expressed transcripts in spleen leukocytes from 2 and 4 week old NOD mice compared to two control strains.

	2 wk (187 genes)		4 wk (327 genes)	
GO category	# of genes in category	Adjusted p-value	# of genes in category	Adjusted p-value
**metabolism**	**84**	**3.78e-6**	**122**	**1.26e-3**
**macromolecule metabolism**	**58**	**9.39e-7**	**76**	**1.10e-3**
**cellular macromolecule metabolism**	**39**	**4.83e-4**	**57**	**1.50e-3**
**protein metabolism**	**42**	**1.82e-4**	**58**	**3.62e-3**
**biopolymer metabolism**	**40**	**1.01e-5**	**52**	**1.03e-3**
**cellular protein metabolism**	**39**	**3.57e-4**	**56**	**1.81e-3**
**protein modification**	**21**	**4.79e-3**	**34**	**7.59e-4**
**biopolymer modification**	**23**	**1.67e-3**	**34**	**1.59e-3**
cellular catabolism	10	4.08e-3	-	-
micromolecule biosynthesis	14	1.83e-3	-	-
cellular carbohydrate metabolism	7	9.99e-3	-	-
biopolymer catabolism	7	4.57e-3	-	-
cellular macromolecule catabolism	8	1.65e-3	-	-
biosynthesis	19	4.66e-3	-	-
amine metabolism	11	1.10e-4	-	-
aminoacid and derivative metabolism	7	9.30e-3	-	-
macromolecule catabolism	9	1.10e-3	-	-
mRNA metabolism	7	2.26e-3	-	-
RNA processing	8	9.03e-3	-	-
chromatin modification	5	8.91e-3	-	-
regulation of biological process	-	-	67	5.83e-3
immune system process	-	-	20	1.50e-3
antigen processing and presentation	-	-	8	1.12e-5
membrane lipid metabolism	-	-	7	4.32e-3
chemotaxis	-	-	7	6.73e-4
actin polymerization and/or depolymerization	-	-	5	4.90e-4
apoptosis	-	-	15	9.15e-3

One-way ANOVA (p<0.05, Benjamini-Hochberg) of mRNA expression data followed by hierarchical clustering identified 187 and 327 probe sets whose expression was altered in spleen leukocytes from NOD mice compared to two control strains (NON and C57BL/6) at 2 weeks and 4 weeks, respectively. The lists of differentially expressed genes were analyzed in WebGestalt (http://bioinfo.vanderbilt.edu/webgestalt) for enriched GO categories using hypergeometric test (p<0.01, Benjamini-Hochberg); dashes (-) indicate that the corresponding categories were not significantly enriched at the respective ages. Categories represented by ≥5 genes are shown. **Bold text** indicates categories that were common to both age groups.

**Table 6:** Enriched molecular function/cellular component Gene Ontology (GO) categories for the lists of differentially expressed transcripts in spleen leukocytes from 2 week and 4 week old NOD mice compared to two control strains.

	2 wk (187 genes)		4 wk (327 genes)	
GO category	# of genes in category	Adjusted p-value	# of genes in category	Adjusted p-value
Molecular function				
**transferase activity**	**29**	**2.54e-3**	**43**	**5.94e-3**
catalytic activity	68	3.65e-5	-	-
transcription factor binding	6	8.06e-3	-	-
nuclease activity	5	9.24e-3	-	-
binding	-	-	167	8.48e-3
protein binding	-	-	84	4.69e-3
zinc ion binding	-	-	42	2.79e-3
sugar binding	-	-	9	3.64e-3
transferase activity, transferring phosphorous containing groups	-	-	29	2.68e-4
kinase activity	-	-	23	4.53e-3
endopeptidase inhibitor activity	-	-	7	9.64e-3
Cellular component				
**intracellular**	**81**	**7.13e-6**	**131**	**8.96e-4**
intracellular membrane-bound organelle	64	2.46e-5	-	-
cytoplasm	41	1.43e-3	-	-
intracellular organelle part	28	6.45e-3	-	-
mitochondrion	15	1.36e-3	-	-
nucleus	-	-	68	8.16e-3
ribonucleoprotein complex	-	-	13	6.21e-3
cell surface	-	-	10	5.97e-5
MHC protein complex	-	-	6	1.76e-5

One-way ANOVA (p<0.05, Benjamini-Hochberg) of mRNA expression data followed by hierarchical clustering identified 187 and 327 probe sets whose expression was altered in spleen leukocytes from NOD mice compared to two control strains (NON and C57BL/6) at 2 weeks and 4 weeks, respectively. The lists of differentially expressed genes were analyzed in WebGestalt (http://bioinfo.vanderbilt.edu/webgestalt) for enriched GO categories using hypergeometric test (p<0.01, Benjamini-Hochberg); dashes (-) indicate that the corresponding categories were not significantly enriched at the respective ages. Categories represented by ≥5 genes are shown. **Bold text** indicates categories that were common to both age groups.

In summary, the GO analyses suggest that spleen leukocytes from NOD mice at the preinsulitis stage have impaired metabolism, which was evident at both 2 and 4 weeks, and impaired immune response and apoptosis, evident only at 4 weeks. The analysis also suggests abnormalities involving the mitochondria in 2 week old NOD mice.

### KEGG pathway enrichment analysis

To identify well characterized molecular pathways that were significantly over-represented in the gene lists of NOD-specific strain differences, we performed KEGG pathway analysis. KEGG pathways are manually drawn maps representing well known molecular interaction and reaction networks. We conducted this analysis also using the expanded transcriptome gene lists described above under GO analysis. The results are shown in Table 7. Consistent with the GO analysis, the predominant category was metabolic pathways, which was highly significantly enriched at 2 weeks (p = 4.98e-8) and slightly less so at 4 weeks (p = 2.65e-5). The lists were also more significantly enriched for pathways involved in RNA splicing (spliceosome) at 2 weeks (p = 2.76e-6) than at 4 weeks (p = 0.0032). Both lists were also enriched for immune function pathways, including natural killer cell mediated cytotoxicity, antigen processing and presentation and T cell receptor signaling. Conversely to the metabolic pathways, the immune function pathways were more significantly enriched at 4 weeks than at 2 weeks. Additionally, each of the lists also had unique significantly enriched pathways, including: at 2 weeks, further metabolic pathways categorizations (such as purine/pyrimidine metabolism, amino sugar and nucleotide sugar metabolism, and amino acid metabolism); and at 4 weeks, immune cell signaling pathways (such as graft-versus-host disease, B cell receptor signaling, cell adhesion molecules, etc) and autoimmune diseases (Type 1 diabetes mellitus and autoimmune thyroid disease). KEGG pathway analysis of the combined list of differentially expressed proteins revealed enrichment for 4 pathways (shown in Table 8) also pointing to the same themes as the transcriptome data.

**Table 7:** Enriched KEGG Pathways for the lists of differentially expressed transcripts in spleen leukocytes from 2 week and 4 week old NOD mice compared to two control strains.

	2 wk		4 wk	
KEGG Pathway	# of genes	Adjusted p-value	# of genes	Adjusted p-value
**Metabolic pathways**	**19**	**4.98e-8**	**21**	**2.65e-5**
**Spliceosome**	**7**	**2.79e-6**	**5**	**0.0032**
**Natural killer cell mediated cytotoxicity**	**4**	**0.0027**	**11**	**5.56e-9**
**T cell receptor signaling**	**5**	**0.0004**	**7**	**5.80e-5**
**Antigen processing and presentation**	**3**	**0.0087**	**7**	**2.35e-5**
Purine metabolism	5	0.0007	-	-
Amino sugar and nucleotide sugar metabolism	3	0.0013	-	-
RNA degradation	3	0.0042	-	-
ECM-receptor interaction	3	0.0059	-	-
Chronic myeloid leukemia	3	0.0059	-	-
Pyrimidine metabolism	3	0.0087	-	-
Glycine, serine and threonine metabolism	2	0.0093	-	-
Graft-versus-host disease	-	-	8	9.04e-8
B cell receptor signaling	-	-	6	5.80e-5
Fc gamma R-mediated phagocytosis	-	-	6	0.0002
Cell adhesion molecules (CAMs)	-	-	7	0.0002
Type I diabetes mellitus	-	-	5	0.0003
Autoimmune thyroid disease	-	-	5	0.0004
Glycosphingolipid biosynthesis – lacto and neolacto series	-	-	3	0.001
Ribosome	-	-	5	0.0023
Complement and coagulation cascades	-	-	4	0.0025
Phosphatidlylinositol signaling system	-	-	4	0.0028
Endocytosis	-	-	6	0.0033
Notch signaling pathway	-	-	3	0.0091
MAPK signaling pathway	-	-	6	0.0099

One-way ANOVA (p<0.05, Benjamini-Hochberg) of mRNA expression data followed by hierarchical clustering identified 187 and 327 genes whose expression was altered in spleen leukocytes from NOD mice compared to two control strains (NON and C57BL/6) at 2 weeks and 4 weeks, respectively. The lists of differentially expressed genes were analyzed in WebGestalt (http://bioinfo.vanderbilt.edu/webgestalt) for enriched KEGG pathways using the hypergeometric test (p<0.01, Benjamini-Hochberg); dashes (-) indicate that the corresponding categories were not significantly enriched at the respective ages. Pathways represented by ≥2 genes are shown. **Bold text** indicates categories that were common to both age groups; ECM, extracellular membrane.

**Table 8:** Enriched KEGG Pathways for the lists of differentially expressed proteins in spleen leukocytes from 2 week and 4 week old NOD mice compared to two control strains.

	2 wk and 4 wk combined (16 proteins)	
KEGG Pathway	# of genes	Adjusted p-value
RNA degradation	2	0.0004
Antigen processing and presentation	2	0.0006
MAPK signaling pathway	2	0.0032
Pathways in cancer	2	0.0036

One-way ANOVA (p<0.05) of protein expression data followed by hierarchical clustering and protein identification revealed 9 and 7 proteins whose expression was altered in spleen leukocytes from NOD mice compared to two control strains (NON and C57BL/6) at 2 weeks and 4 weeks of age, respectively. The two lists of differentially expressed proteins were combined and protein identifiers (IDs) were converted to gene IDs. Gene IDs were uploaded and analyzed in WebGestalt (http://bioinfo.vanderbilt.edu/webgestalt) for enriched KEGG pathways using the hypergeometric test (p<0.01, Benjamini-Hochberg). Pathways represented by ≥2 proteins are shown.

Overall, the KEGG pathway analyses suggest that two broad molecular pathways – metabolic and immune response pathways – are altered in the various immune cell types (natural killer cells, antigen presenting cells (B cells, macrophages, dendritic cells) and T cells (CD4 and CD8 T-cells). Alterations in the metabolic pathways appear to be predominant at 2 weeks, while those in the immune response and signaling pathways appear to be predominant at 4 weeks. Thus, the data suggest that impairment of the metabolic pathways may be the primary defect contributing to initiation of autoimmune pathology in NOD mice.

### Ingenuity pathway analysis

**Figure 3:**
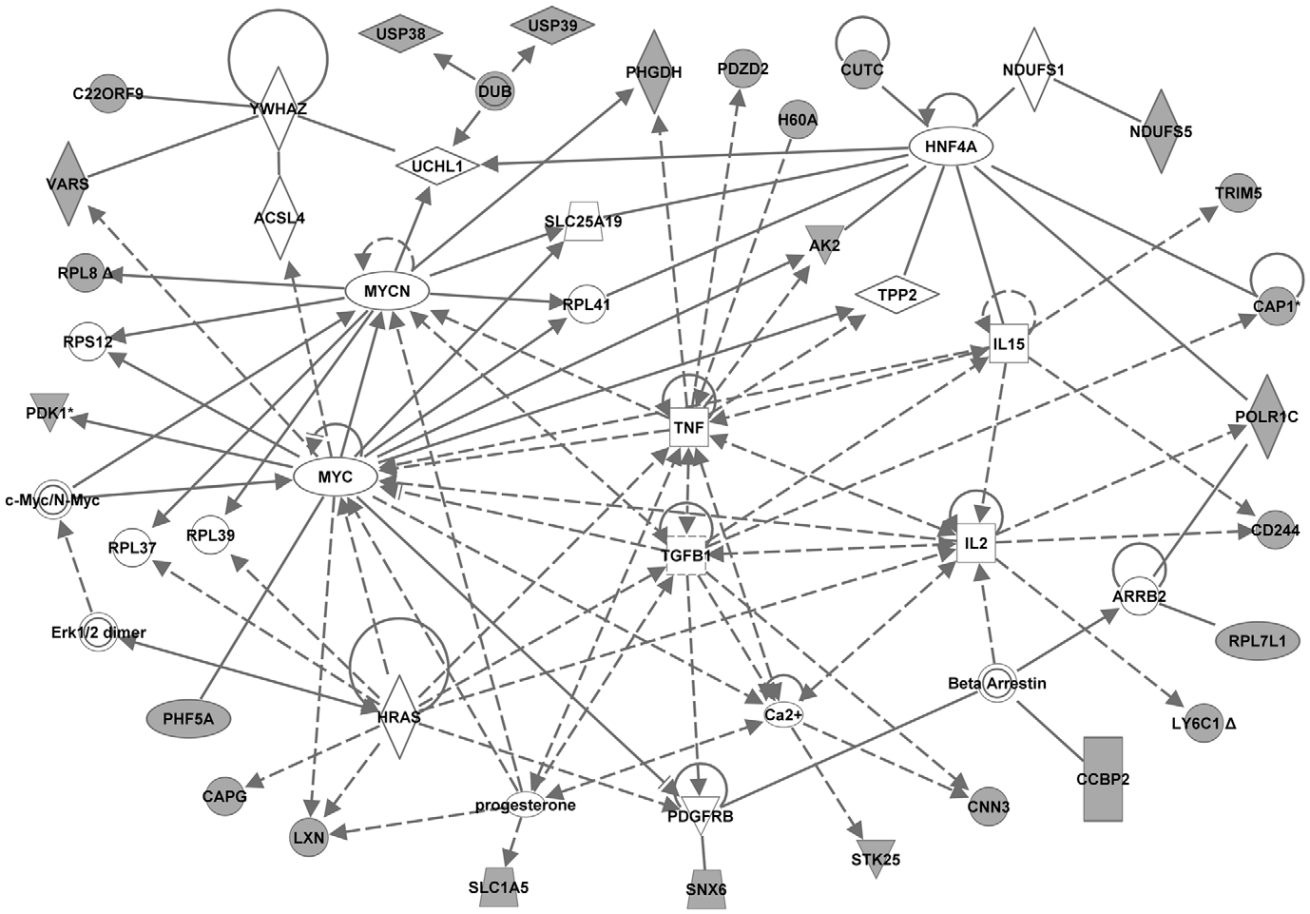
Transcriptome network created by Ingenuity Pathway Analysis from the dataset of 2 week-old mice. The merged network was generated from the list of transcripts differentially expressed in 2 week-old NOD mice compared to both control strains, NON and C57BL/6. The list was selected from a hierarchical cluster of 175 genes that had highly significant expression differences between strains at 2 weeks. The genes derived from our uploaded gene list (focus genes) are represented by gray icons. White icons represent genes (or endogenous chemicals) derived solely from the IPA knowledge base and that could be algorithmically connected to the focus genes.

**Figure 4:**
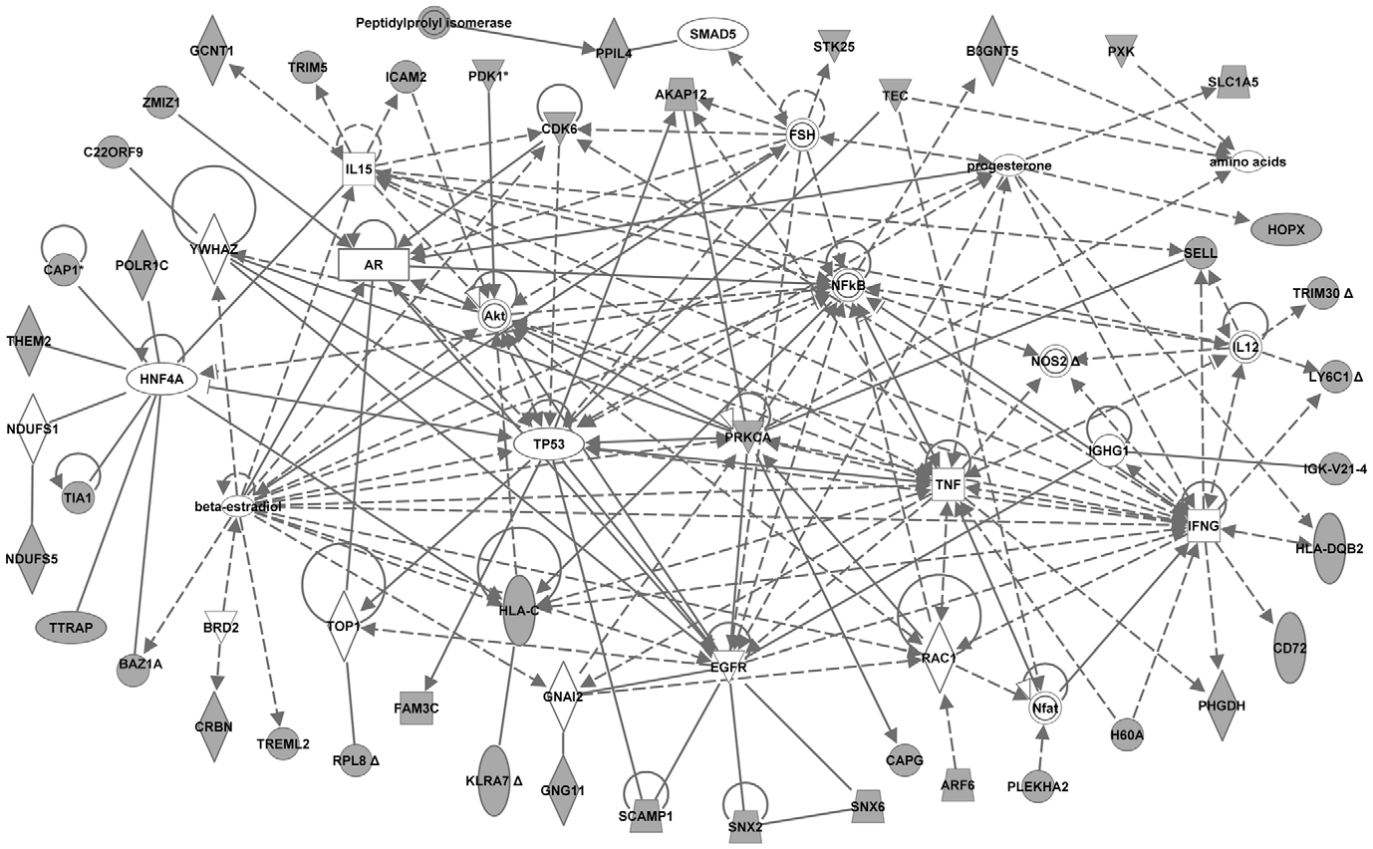
Transcriptome network created by Ingenuity Pathway Analysis from the dataset of 4 week-old mice. The merged network was generated from the list of transcripts differentially expressed in 4 week-old NOD mice compared to both control strains, NON and C57BL/6. The list was selected from a hierarchical cluster of 189 genes that had highly significant expression differences between strains at 4 weeks. The genes derived from our uploaded gene list (focus genes) are represented by gray icons. White icons represent genes (or endogenous chemicals) derived solely from the IPA knowledge base and that could be algorithmically connected to the focus genes.

**Figure 5:**
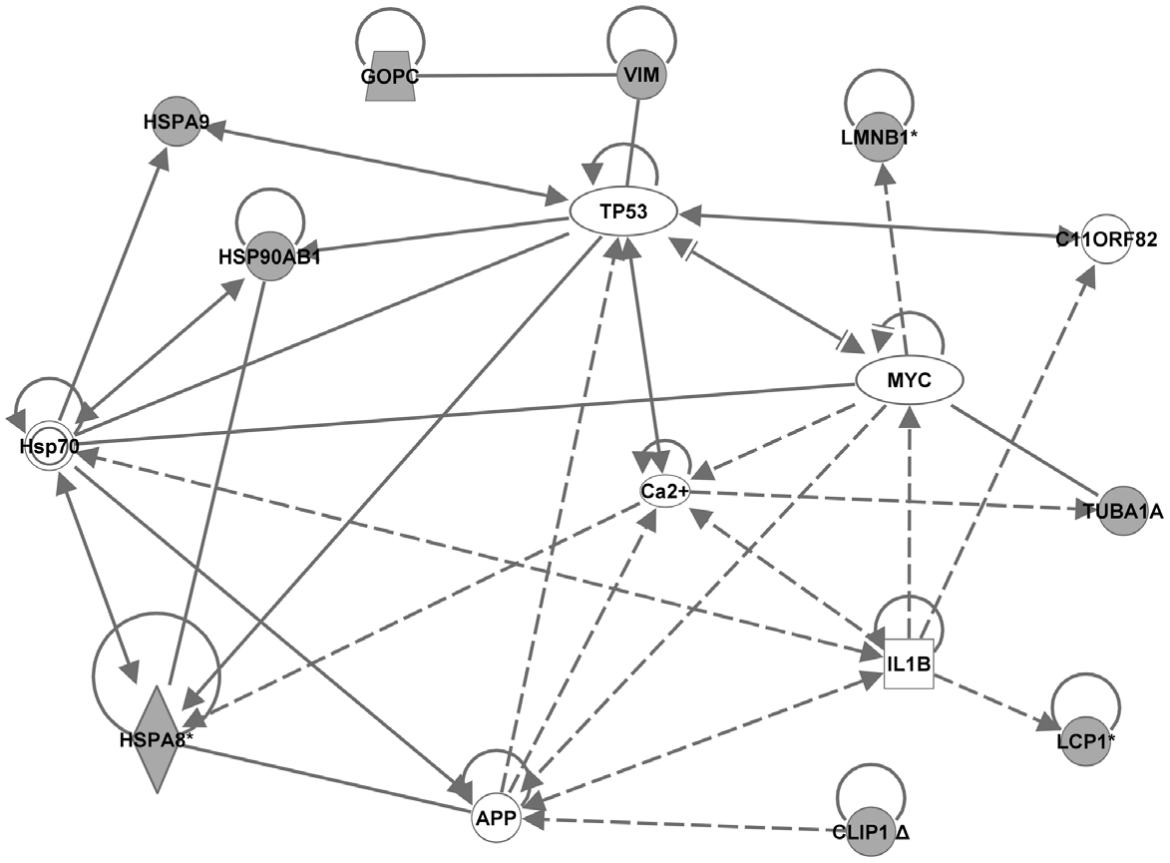
Proteome network created by Ingenuity Pathway Analysis from the dataset of 2 week-old mice. A single network was generated from the list of proteins that were differentially expressed in 2 week-old NOD mice compared to both control strains, NON and C57BL/6. The protein spots from which these proteins were identified were selected from the hierarchical cluster of 19 protein spots that had significant expression differences between strains at 2 weeks. The gene names derived from our uploaded lists (focus genes) are represented by gray icons. White icons represent genes (or endogenous chemicals) derived solely from the IPA knowledge base and that could be algorithmically connected to the focus genes.

**Figure 6:**
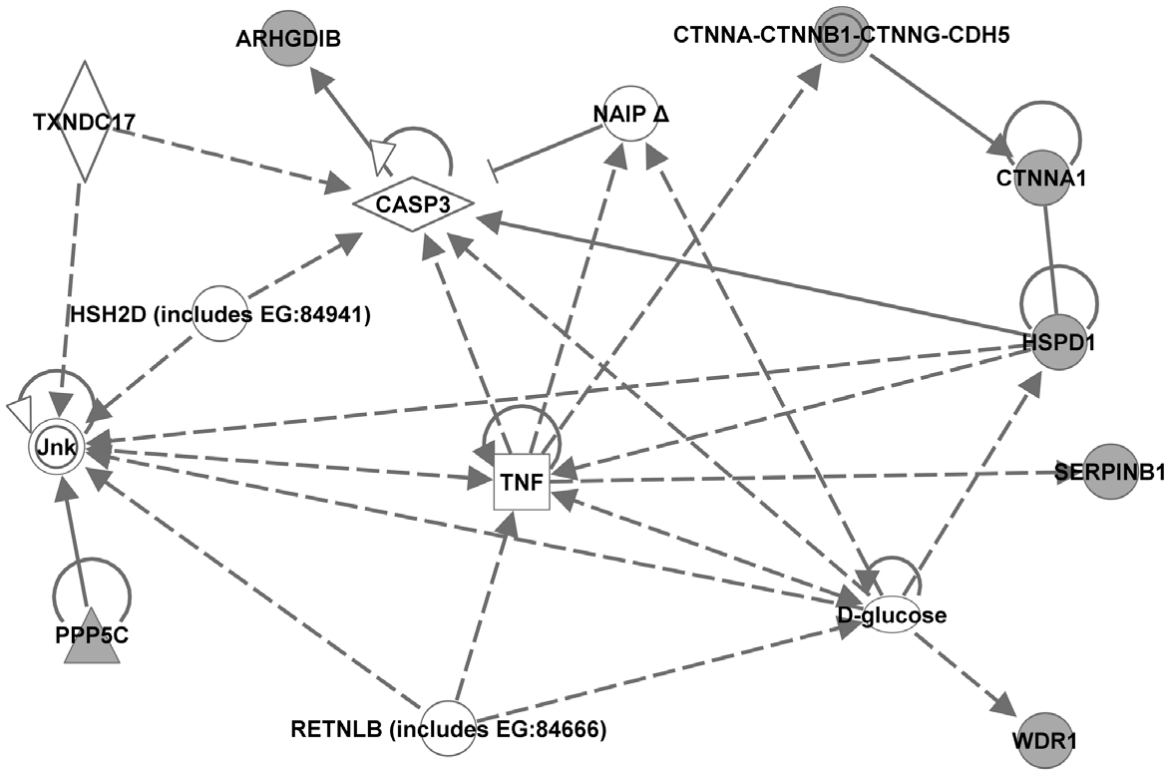
Proteome network created by Ingenuity Pathway Analysis from the dataset of 4 week-old mice. A single network was generated from the list of proteins that were differentially expressed in 4 week-old NOD mice compared to both control strains, NON and C57BL/6. The protein spots from which these proteins were identified were selected from the hierarchical cluster of 26 protein spots that had significant expression differences between strains at 4 weeks. The gene names derived from our uploaded lists (focus genes) are represented by gray icons. White icons represent genes (or endogenous chemicals) derived solely from the IPA knowledge base and that could be algorithmically connected to the focus genes.

To identify the genes and/or endogenous chemicals that may play a role in regulating the abnormal molecular pathways in NOD mice, we performed Ingenuity pathway analysis (IPA). IPA generates *de novo* networks from the uploaded gene lists based on information from the literature contained in the IPA knowledge base (see “Materials and Methods” for details). IPA analyses of the lists of NOD-specific strain differences derived at the most stringent threshold (Benjamini-Hochberg, p<0.005; Tables 1 and 2) generated several networks each. Each respective set of networks were merged to create one molecular network for each age (Figure 3 and Figure 4, for 2 and 4 weeks, respectively). The transcriptome network for the 2 week old mice dataset clustered around several central genes including MYC/MYCN, HNF4A, YWHAZ, TNF, IL2, IL15, TGFB1, progesterone, and Ca2+. That for the 4 week old mice dataset also clustered around several genes including TP53, YWHAZ, HNF4A, TNF, IFNG, IL15, beta-estradiol, progesterone, Akt, PRKCA, IL12, and HLA-C. IPA analyses of the lists of NOD differentially expressed proteins at 2 weeks (9 proteins) and at 4 weeks (7 proteins) generated one network each. The proteome network for the 2 week old mice centered around TP53, MYC, APP, IL1B, Ca2+ and HSPA8 (Figure 5). That for the 4 week-old NOD mice clustered around TNF, CASP3, HSPD1, D-glucose and Jnk (Figure 6). The major biological functions significantly represented by the transcriptome networks included cancer (indicative of apoptosis/cellular proliferation) and immunological disease/response (Table 9). The top IPA functional categories represented by the proteome networks were cell proliferation, cell death and oxidative stress (Table 9). To facilitate an objective comparison between networks and to enable us gain further insights into the genes that may play a central role in each network, we ranked the central genes according to the total number of connections linked to each gene (Table 10). This analysis revealed that five central genes (TNF, HNF4A, IL15, Progesterone, and YWHAZ) were common to the transcriptome networks. A greater number of signaling and/or immune response genes (such as IFNG, EGFR, NFKB, Akt, PKRCA, IL12, and HLA-C) were present in the transcriptome network for 4 week-old mice than in that of 2 week-old mice. Inspection of the central genes between the proteome networks indicated that none were shared between the two networks (Table 10). However, a few molecules (TP53, MYC and Ca2+) were shared between the transcriptome and proteome networks of the 2 week old mice. Moreover, TNF was central to three networks, both the transcriptome networks and the proteome network for the 4 week-old mice.

**Table 9:** Functional categories associated with the major Ingenuity Pathway Networks of differentially expressed transcripts/proteins in spleen leukocytes from 2 week and 4 week old NOD mice.

Network	# of focus genes^a^	Top IPA functional categories
**Transcriptome – 2 week**		
1	13	cancer, cell morphology, immunological disease
2	13	cell morphology, cellular development, hematological system development and function
**Transcriptome – 4 week**		
1	17	developmental disorder, cancer, cell death
2	13	cancer, cell cycle, gastrointestinal disease
3	12	cellular development, drug metabolism, lipid metabolism
**Proteome – 2 week**		
1	9	free radical scavenging, molecular transport, cancer
**Proteome – 4 week**		
1	6	cell death, cancer, cell morphology

One-way ANOVA of expression data sets followed by hierarchical clustering identified transcripts/proteins differentially expressed in spleen leukocytes from NOD mice compared to two control strains (NON and C57BL/6). The lists of differentially expressed genes were analyzed by Ingenuity Pathway Analysis (www.ingenuity.com).

a Genes that were present in the uploaded lists.

**Table 10:** Central Genes in the Ingenuity Pathway Networks of differentially expressed transcripts/proteins in spleen leukocytes of 2 and 4 week old NOD mice.

Central Gene	Total Connections (# of Focus genes^a^)	Focus Genes^a^
**Transcriptome – 2 week**		
MYC	20 (5)	**PDK1**,AK2, LXN, PHF5A, VARS
**TNF**	13 (4)	**H60A**, **PHGDH**, AK2, PDZD2
MYCN	13 (2)	**RPL8**, PHGDH
TGFB1	11 (2)	**CAP1**, CNN3
**HNF4A**	10 (4)	**CAP1**, **POLR1C**, AK2, CUTC,
IL2	10 (3)	**LY6C1, POLR1C**, CD244
HRAS	10 (2)	**CAPG**, LXN,
**IL15**	7 (2)	**TRIM5**, CD244
**Progesterone**	7 (2)	**SLC1A5**, LXN
Ca2+	7 (2)	**STK25**, CNN3,
PDGFRB	5 (1)	**SNX6**
**YWHAZ**	4 (2)	**C22ORF9**, VARS
**Transcriptome – 4 week**		
IFNG	22 (9)	**H60A**, **LY6C1**, **PHGDH**, CDK6, CD72, HLA-DQB2, HLA-C, PRKCA, SELL
Beta-estradiol	19 (5)	BAZ1A, CDK6, HLA-C, PRKCA, TREML2
**TNF**	18 (5)	**H60A**, **PHGDH**, AKAP12, HLA-C, PRKCA
TP53	18 (6)	AKAP12, CDK6, FAM3C, PRKCA, SCAMP1, TEC
EGFR	17 (4)	**SNX6**, PRKCA, SCAMP1, SNX2,
NFKB	16 (3)	B3GNT5, HLA-C, PRKCA
Akt	15 (4)	**PDK1**, HLA-C, ICAM2, PRKCA
PRKCA^a^	14 (4)	AKAP12, **CAPG**, PRKCA, SELL
**IL15**	12 (5)	**TRIM5**, CDK6, ICAM2, GCNT1, SELL
**HNF4A**	11 (7)	**CAP1**, **POLR1C**, BAZ1A, HLA-C, THEM2, TIA1, TTRAP
FSH	11 (3)	**STK25**, AKAP12, CDK6
**Progesterone**	10 (3)	**SLC1A5**, HLA-DQB2, HOPX
AR	10 (2)	CDK6, ZMIZ1
IL12	8 (3)	LY6C1, SELL, TRIM30
HLA-C^a^	7 (2)	HLA-C, KLRA7
**YWHAZ**	6 (2)	**C22ORF9**, PRKCA
CDK6 (FG)	6 (1)	CDK6
Amino acids	4 (4)	B3GNT5, PRKCA, PXK, TEC
**Proteome – 2 week**		
TP53	9 (4)	HSPA8, HSPA9, HSP90AB1, VIM
MYC	7 (2)	LMNB1, TUBA1A
APP	7 (2)	CLIP1, HSPA8
Ca2+	6 (2)	HSPA8, TUBA1
IL1B	6 (1)	LCP1
HSPA8^a^	5 (2)	HSPA8, HSP90AB1
**Proteome – 4 week**		
TNF	8 (2)	CTNNA, HSPD1
CAPS3	7 (2)	ARHGDI, HSPD1
D-glucose	7 (2)	HSPD1, WDR1
Jnk	7 (2)	HSPD1, PPP5C
HSPD1^a^	5 (2)	CTNNA1, HSPD1

a Genes that came from the list of differentially expressed genes in NOD mice compared to control strains (NON and C57BL/6). The connections to each gene (i.e. edges representing gene-gene relationships) in the molecular networks were manually counted. The central genes were then ranked from highest to lowest based on the number of total connections; cut-off, 4 total connections. Genes indicated in **Bold text** are central or focus genes that were common to both ages.

Taken together, IPA analyses of networks generated from NOD-specific strain differences identified several key factors that may play a role in regulating the molecular pathways that are abnormal in spleen leukocytes from NOD mice at the preinsulitis stage of autoimmune diabetes.

Because the gene list of NOD-specific age differences (Table 3) was suggestive of immune activation, we conducted IPA on the three lists of strain-specific age differences (Tables 3, S2 and S3). The functional categories associated with the three topmost major networks generated from each of these lists are shown in Table 11. Only the NOD networks are associated with immunological disease and inflammatory response. The three networks for each strain were merged to create one molecular network for each strain (Figures S4, S5 and S6 for NOD, NON and C57BL/6, respectively). The network for NOD age differences centered around several cytokines/cytokine receptors (IFNG, IL12, interferon alpha, IL17RB) and acute phase signaling molecules (PI3K, Akt, ERK, p38MAPK, NFKB). Interestingly, the network of NON-specific age differences also had IFNG as a central gene (but lacked the other cytokines observed in the NOD network) and only NFKB of the acute phase signaling molecules seen in the NOD network. However, the NON network also had TGFB, STAT1 and Ca2+ as central molecules. Strikingly, the network generated from the C57BL/6-specific age differences conspicuously lacked the cytokines seen in the NOD network (except interferon alpha) but had all the acute phase signaling molecules seen in the NOD network. In summary, in NOD mice, ∼60% of the differentially expressed genes are upregulated at 4 weeks as compared to at 2 weeks, and a vast majority of these genes appear to be involved in the acute phase signaling cascade (PI3K, Akt, ERK, p38MAPK, NFKB, Figure S4), possibly driven by IFNG and other cytokines. In contrast, in C57BL/6 mice, ∼75% of the differentially expressed genes are downregulated at 4 weeks compared to at 2 weeks. Intriguingly, a large majority of these genes are also involved in the acute phase signaling cascade (Figure S6). These results would suggest that the acute phase signaling pathway is activated in the NOD mice (inasmuch as the majority of the genes are upregulated) whereas it is suppressed in the C57BL/6 mice (because the majority of the genes are downregulated at 4 weeks). Thus, the data suggest immune activation in the NOD mice between 2 weeks and 4 weeks possibly driven by the inflammatory cytokine IFNG.

**Table 11:** Functional categories associated with the 3 topmost major IPA Networks of transcripts uniquely differentially expressed in spleen leukocytes between 2 weeks and 4 weeks of age in NOD, NON and C57BL/6 mice.

Network	# of focus genes^a^	Top IPA functional categories
**NOD**		
1	15	cellular function and maintenance, immunological disease
2	13	cell death, DNA replication, recombination, repair, gene expression
3	10	inflammatory response, renal/urological disease, infectious disease
**NON**		
1	22	hematological system development and function, tissue morphology, cell-mediated immune response
2	20	cell cycle, cancer, skeletal and muscular disorders
3	17	cellular assembly and organization, cell morphology and cellular development
**C57BL/6**		
1	19	cellular morphology, cellular assembly and organization, cellular function and maintenance
2	17	cellular movement, immune cell trafficking, cell-to-cell signaling and interaction
3	16	post-translational modification, cell cycle, cellular assembly and organization

Statistical analysis of mRNA expression data by unpaired t-Test (p<0.001) followed by Venn diagram analysis identified transcripts uniquely differentially expressed in spleen leukocytes between 2 weeks and 4 weeks of age in NOD mice and two control strains, NON and C57BL/6. The lists of differentially expressed genes were analyzed by IPA (Ingenuity Pathway Analysis; www.ingenuity.com).

a Genes that were present in the uploaded lists.

## Discussion

Insulitis, the earliest sign of autoimmune pathology in the NOD mouse model for autoimmune (Type 1) diabetes, is first obvious at around 4–5 weeks of age [1]. The purpose of our study was to gain new insights into the molecular events that lead to this process and that may influence initiation of autoimmune diabetes. Since autoimmune diabetes is a multigenic disease, the present study tested the hypothesis that alterations in multiple molecular pathways lead to initiation of autoimmune pathology. We conducted a detailed analysis of genome-wide gene expression abnormalities in the spleen leukocytes (i.e. the effector tissue) of young NOD mice in the preinsulitis stage of autoimmune diabetes at ages 2 and 4 weeks. This study differs from our previous efforts [14] in that gene expression in the NOD mouse was compared to two controls (NON and C57BL/6) instead of one control (C57BL/6). This allowed us to eliminate C57BL/6-specific differences (i.e. C57 low and C57 high, Figure 1 and 2) and to focus on only those genes where NOD expression differed from that of C57BL/6 and an additional control (NON); thus, increasing the stringency of our attempt at identifying diabetes-related abnormalities. Additionally, we conducted a 1-w ANOVA in the current study instead of a 2-w ANOVA that we performed in the previous study [14]. This allowed us to put a stronger focus on differences unique to each specific age, a significant number of which could have been excluded by a 2-w ANOVA. Furthermore, we applied a modern suite of bioinformatics approaches to gain the fullest possible insights from these datasets. The current study confirms the results of our previous study, which showed a defect in apoptosis/cell proliferation. Furthermore, it provides new insights into the molecular events that occur prior to insulitis in a time course fashion. Thus, we identified abnormalities in two broad pathways: metabolic, which were predominant at 2 weeks, and immune response pathways, which were predominant at 4 weeks.

Surprisingly, analysis of the global gene expression profiles and hierarchical clustering revealed that the vast majority (∼90%) of the abnormally expressed genes in NOD spleen leukocytes compared to two control strains were repressed. A previous study [23] also investigating gene expression changes in various tissues (pancreatic lymph nodes, spleen and peripheral blood cells) and at various ages (10 days, 4 weeks, 12 weeks, 16 weeks, or 20 weeks) in NOD mice compared to NOD.B10 mice (in which a non-permissive MHC haplotype is imposed onto NOD to silence autoimmune disease) also reported that the great majority of identified NOD differentially expressed genes were down regulated (except only with peripheral blood cells at 4 weeks). It is important to note that, unlike our study, this study did not identify any differentially expressed genes in the spleen tissue at ages 2 weeks and 4 weeks. This could be due to their use of the whole spleen tissue rather than the leukocyte fraction as we did in our study and/or their narrowing changes down to only the diabetes susceptibility genes in the MHC region (excluding differences influenced by all the other chromosomal regions conferring diabetes susceptibility). Indeed, the authors noted that accurate identification and characterization of genes involved in complex autoimmune diseases like T1D may also require analysis of expression in specific cells of the tissues.

Gene ontology (GO) analyses of the NOD differentially expressed genes identified in our study revealed alterations in several processes that were common to both ages (Tables 5 and 6), including abnormalities in metabolism and enzymatic activity. Abnormalities in genes involved in metabolism (including the mitochondrion, carbohydrate and amine metabolism) and biosynthesis were predominant at 2 weeks of age while abnormalities in the immune system process (including response to external stimulus, antigen processing and presentation) were predominant at 4 weeks of age. KEGG pathway analysis (Tables 7 and 8), likewise, identified that abnormalities in metabolic pathways were predominantly enriched at 2 weeks, including spliceosome, purine/pyrimidine metabolism, amino acid and sugar metabolism. Alterations in the immune system function and signaling pathways including natural killer cell mediated cytotoxicity, antigen processing and presentation, B and T cell receptor signaling, graft-versus-host disease, Fc gamma R-mediated phagocytosis, type 1 diabetes mellitus, etc. were predominantly enriched at 4 weeks.

Spleen leukocytes are naturally in a resting state with low level of protein synthesis until they encounter an activation signal, which leads to rapid growth followed by proliferation. This immune activation results in a dramatic demand for energy and biosynthetic precursors that is met by upregulation of metabolism. Because metabolism was a significantly enriched process in the NOD differentially expressed genes, yet the vast majority of these genes were repressed, we hypothesize that metabolism is repressed in NOD mice as compared to the two control strains. Pre-autoimmune metabolic changes have been previously reported in female NOD mice that later progressed to autoimmune diabetes [24]. Dysregulation of lipid and amino acid metabolism was observed in these mice. Interestingly, dysregulation of lipid and amino acid metabolism has also been observed in the serum of children who later progressed to human type 1 diabetes in the period prior to seroconversion to islet autoantibody positivity [25]. Thus, NOD autoimmune diabetes and human T1D may have some similarities in this respect. Furthermore, it should be noted that the elimination of autoreactive lymphocytes is normally done by apoptosis, an active process that requires ATP provided by metabolism.

Significantly enriched immune response pathways that we identified are consistent with the idea of an activation of immune system activity. However, we think that this process is repressed in NOD mice as compared to the two control strains because the vast majority of NOD differentially expressed genes were repressed. As stated above, a previous study also investigating gene expression changes in NOD mice [23] found the same result. However, they did not pursue this observation any further in their report. It is interesting to note, however, that many of the genes identified in this study were also found within the previously identified diabetes susceptibility regions, the *Idd* chromosomal loci. We think that the idea of an apparent paradoxical immune suppression in NOD mice may help explain previous observations that have been made in these mice: i) that immunosuppression exacerbates autoimmune diabetes in NOD mice [26]; and conversely, ii) that immunostimulation circumvents diabetes in NOD mice [27]. Notwithstanding that NOD mice have important differences in their immune system compared to humans, this idea of paradoxical immune suppression appears to have support from human type 1 diabetes studies [28], [29]. Elo et al. [28] found a high-level of repression of immune response genes (including MHC Class I and Class II molecules, T-cell receptor signaling, actin filament system and NF-kB signaling) in children that had developed T1D-associated autoantibodies (prediabetes) who eventually progressed to clinical T1D. Similar to our study, they also found evidence of impairment of transcription, transport, metabolism and cell proliferation. Additionally, another study found a repression of all genes that were differentially expressed in whole untreated CD4 T-cells from new onset T1D patients compared to matched controls [29]. This repression affected key immune functions, such as immune signaling pathways, and cell cycle regulation. In functional studies, these authors found that CD4 T-cell surface expression levels of two key adhesion molecules, LFA-1 and P-selectin, were significantly lower in T1D patients compared to controls. Furthermore, this study demonstrated that a lower percentage of CD4 T-cells from patients entered into mitosis as compared to those from controls suggesting inhibition of CD4 T-cell proliferation in T1D patients. They concluded that CD4 T-cells in new onset T1D have a hyporesponsive immune cell phenotype.

Whereas a generalized repression of the immune system in autoimmune diabetes may seem counterintuitive in a disease caused by activation of the immune system against target self antigens, it should be remembered that tolerance to self antigens requires the activation of self-reactive lymphocytes and their elimination by apoptosis as a result of this activation. Furthermore, elimination of self-reactivity by lymphocytes in the periphery also requires activation of regulatory mechanisms (such as regulatory T cells). Our data are consistent with the hypothesis that a defect in the mitochondria (Table 6) of NOD spleen leukocytes coupled with impaired metabolism leads to a defect in antigen processing/presentation and T cell stimulation (Tables 5, 6, 7, 8), ultimately resulting into generalized immune suppression. Islet-specific autoimmunity likely occurs due to defective tolerance mechanisms in the NOD mice either failing to eliminate islet-reactive T cells by apoptosis or neutralize them by regulatory T-cells activity. To this end, it is worth noting that some of the identified NOD differentially expressed genes (Ly6c1, Trim 12, and Trim30) are known to be linked to a defect in T cell negative selection. Furthermore, the MHC class I-related glycoprotein H60 (H60a), which we identified as being highly expressed in NOD, but virtually unexpressed in control strains (Tables 1 and 2; Figure S1), is known to play a role in immune regulatory mechanisms. H60a can mediate strong suppressive effects on T cell proliferation [30]. It has also been shown to play a role in NK cell tolerance in experimental autoimmune encephalomyelitis [31]. Additionally, a recent study, using a mouse model of spontaneous autoimmune arthritis [32], provided evidence suggesting that efficient suppression of autoimmune diseases may require polyclonal specificities rather than single antigen-specific regulatory T cell activity.

Network analysis by IPA identified several genes/molecules (central genes, Table 10) that we think may play a key role in regulating the identified abnormal processes. Thus, five central genes common to both transcriptome networks were identified, including the proinflammatory cytokine, TNF, a transcription factor (HNF4A), a cytokine (IL15) and two cellular activation/signaling molecules (YWHAZ and progesterone). In addition to the common central genes, unique central genes were identified in the respective networks, e.g. cytokines IL2 and TGFB1, and transcription factors MYC/MYCN in 2 week transcriptome network; and cytokines IFNG and IL12, transcription factors/regulators beta-estradiol, TP53, NFKB, and AR, and cellular activation/signaling EGFR, Akt, PRKCA in 4 week transcriptome network. Although, several of these genes have been previously known to be associated with T1D development, and with immune function, many are novel.

TNF-α, a proinflammatory, multifunctional cytokine was present in three of the four networks underscoring its importance in the initiation of autoimmune diabetes. TNF-α in synergy with IFN-γ (which was present in the 4 week transcriptome network) is known to be important in inducing autoimmunity in the developing immune system [33]–[35]. Also, another report indicated that progression to insulitis in the NOD mouse was connected to changes in T cell activation and signaling associated with TNF-α and another proinflammatory cytokine, IL-6 [36]. Our identification of IL2 as a central gene (at 2 weeks) further supports involvement of IL2 signaling in T1D pathogenesis. The defect in IL2 may contribute to the impairment of regulatory T-cells [37]. The NFKB pathways are essential for the function of antigen presenting cells and related defects have previously been reported in T1D patients [38]. The pregnancy hormones, beta-estradiol and progesterone are known to lead to improvement in T1D in some patients as evidenced by a decline in insulin requirement during pregnancy [39], [40]. Estrogens are known to play a role in modulating immune function of various immune cells and may also play a role in regulating glucose homeostasis and plasma lipids [41]. Progesterone is known to modulate macrophage activation [42].

Myc, an important factor in regulating apoptosis and cellular growth, was central in both the transcriptome and proteome based networks of the 2 week old mice. It has been hypothesized that defects in the process of apoptosis might explain why autoreactive leukocytes persist in the NOD mouse [43]–[45]. Indeed, upregulation of Myc in lymphocytes from NOD mice (in contrast to Myc downregulation in C57BL/6) was associated with their resistance to glucocorticoid-induced apoptosis [46]. Additionally, NOD mice treated with the streptococcal wall component OK432, which restores dexamethasone-induced apoptosis and is associated with downregulation of Myc, do not develop diabetes. Recently, Wang et al. [47] revealed a novel function of Myc as an essential regulator of activation-induced T cell metabolic reprogramming. T cell activation (and other immune cells) leads to a metabolic switch to increased glucose and glutamine metabolism. These authors showed that Myc plays a key role in upregulation of glucose, glutamine, and polyamine metabolism and that its deficiency prevented T-cell proliferation. In addition to Myc as a metabolic regulator, the Akt-mTOR (mammalian target of rapamycin) pathway also promotes posttranslational regulation of glycolytic metabolism [48]. Akt was central in the transcriptome network at 4 weeks.

In addition to the above components, our analysis identified components previously unrecognized to play a role in immunological function, including, p53, HNF4A, and AR (androgen receptor). Tumor suppressor p53 is widely known to act in response to excessive stresses and abnormalities in cell physiology. Recent studies, however, suggest that p53 has additional important roles in normal cellular physiology [49]. To this end, the p53 pathway is tightly involved in the homeostatic regulation of metabolic processes. In these novel functions, p53 helps to align metabolic processes with the proliferation and energy status of the cells, to maintain optimal mode of glucose metabolism and to boost the energy efficient mitochondrial respiration in response to ATP deficiency. Additionally, under normal physiological conditions, p53 mobilizes defenses against “physiological” levels of reactive oxygen species (ROS) to maintain homeostasis. Thus, a possible hypothesis would be that the novel functions of p53 play a role in the physiological immunological process of immune cell activation in response to stimuli, including antigenic stimulation. Our data suggest that these p53 functions are abnormal in NOD mice. HNF4A, a nuclear receptor transcription factor, has been linked to developmental and metabolic functions, and to several diseases, including maturity-onset diabetes of the young and type 2 diabetes [50]. Although HNF4A is known to be expressed in the spleen (among several other tissues), its role in the immunological process has yet to be fully established [51]. Understanding this role may be important as HNF4A could be a good candidate for a drug target. Indeed, this idea has been further reinforced by the recent identification of linoleic acid as the long-sought for ligand for HNF4A [52]. HNF4A controls the expression of numerous genes, including well known target genes involved in regulating cellular metabolism and homeostasis, as well as many new direct target genes involved in regulation of the cell cycle and immune function [53], [54]. AR is a nuclear receptor transcription factor involved in the regulation of many different physiological processes, including developmental, immunological, metabolic and homeostatic processes [55]–[59]. Classical AR signaling occurs via binding of androgens to the AR, which then translocates to the nucleus and activates transcription leading to growth promotion and differentiation. However, AR can also be activated in an androgen-independent fashion via growth factors or cytokines. AR dysfunction is associated with a wide variety of pathologies including prostate cancer, type 1 diabetes and metabolic disorders, such as type 2 diabetes.

In summary, our study identified abnormal molecular pathways in the spleen leukocytes from young NOD mice in the preinsulitis (i.e. early inductive) stage of autoimmune diabetes. The pathways associated with the NOD-specific strain differences can be divided broadly into 2 categories, metabolic pathways (predominant at 2 weeks) and immune response pathways (predominant at 4 weeks). The identified putative key regulators of the metabolic pathways include Myc and HNF4A at 2 weeks, and beta-estradiol, p53, Akt, HNF4A and AR at 4 weeks. The molecular pathways associated with NOD-specific expression differences between 2 week old and 4 week old mice suggest an immune activation phenotype in these mice. We hypothesize that the NOD-specific strain differences we identified represent a defect in immune tolerance predisposing NOD mice to autoimmune diabetes development while the age differences represent the additional changes that accompany initiation of insulitis. It is worth noting that since spleen leukocytes constitute a heterogeneous cell population the molecular networks identified by IPA are likely representative of the predominant cell types. Thus, future experiments to dissect altered molecular networks within individual cell types are warranted. Indeed such studies may reveal age-specific gene expression abnormalities that could have been masked in the current study. Notwithstanding, our data on untreated unfractionated spleen leukocytes suggest that that abnormalities in regulation of metabolic pathways in the immune cells of young NOD mice lead to abnormalities in the immune response pathways and as such may play a role in the initiation of autoimmune diabetes. Indeed our study suggests that targeting metabolism may be a novel strategy to modulate autoimmunity and to prevent and/or treat autoimmune diabetes.

## Supporting Information

Figure S1 **Symbols in Ingenuity Pathway Analysis networks and their meaning.**
(PPT)

Figure S2 **Validation of microarray data by quantitative real-time PCR.** Quantitative real-time PCR was performed for H60a, Trim5, Trim12, Ly6c1, Snx6 and Sp110. Fold change values represent expression levels of genes in control strains (NON and C57BL/6 mice) relative to NOD mice. The results are consistent with the microarray data results.
(PPT)

Figure S3 **Sample 2D gel images of protein extracts from spleen leukocytes of one control strain (C57BL/6, A) and NOD mice (B).** The approximate pI and molecular mass in kDa is given on the x and y axes, respectively.
(PPT)

Figure S4 **Transcriptome network created by Ingenuity Pathway Analysis from genes uniquely differentially expressed in NOD mice between 2 weeks and 4 weeks of age.** The merged network was generated from the list of transcripts uniquely differentially expressed in spleen leukocytes of NOD mice between 2 weeks and 4 weeks of age in comparison to two control strains, NON and C57BL/6. It represents the three topmost major networks. The genes derived from our uploaded gene list (focus genes) are represented by gray icons. White icons represent genes (or endogenous chemicals) derived solely from the IPA knowledge base and that could be algorithmically connected to the focus genes.
(TIFF)

Figure S5 **Transcriptome network created by Ingenuity Pathway Analysis from genes uniquely differentially expressed in NON mice between 2 weeks and 4 weeks of age.** The merged network was generated from the list of transcripts uniquely differentially expressed in spleen leukocytes of NON mice between 2 weeks and 4 weeks of age in comparison to NOD and C57BL/6. It represents the three topmost major networks. The genes derived from our uploaded gene list (focus genes) are represented by gray icons. White icons represent genes (or endogenous chemicals) derived solely from the IPA knowledge base and that could be algorithmically connected to the focus genes.
(TIFF)

Figure S6 **Transcriptome network created by Ingenuity Pathway Analysis from genes uniquely differentially expressed in C57BL/6 mice between 2 weeks and 4 weeks of age.** The merged network was generated from the list of transcripts uniquely differentially expressed in spleen leukocytes of C57BL/6 mice between 2 weeks and 4 weeks of age in comparison to NOD and NON mice. It represents the three topmost major networks. The genes derived from our uploaded gene list (focus genes) are represented by gray icons. White icons represent genes (or endogenous chemicals) derived solely from the IPA knowledge base and that could be algorithmically connected to the focus genes.
(TIFF)

Table S1 **Genes and primers/probes used for quantitative Real-time PCR.**
(DOC)

Table S2 **Transcripts significantly differentially expressed in spleen leukocytes between 2 weeks and 4 weeks of age only in NON mice compared to NOD and C57BL/6 mice, and their p-values and fold changes.**
(XLS)

Table S3 **Transcripts significantly differentially expressed in spleen leukocytes between 2 weeks and 4 weeks of age only in C57BL/6 mice compared to NOD and NON mice, and their p-values and fold changes.**
(XLS)

Table S4 **Transcripts significantly differentially expressed in spleen leukocytes of 2 week old NOD mice with their ANOVA p-values (Benjamini-Hochberg, p<0.05) and fold-changes relative to two control strains, NON and C57BL/6.** A. 162 probesets of significantly lower expression in NOD mice; and B. 25 probesets of significantly higher expression in NOD mice.
(XLS)

Table S5 **Transcripts significantly differentially expressed in spleen leukocytes of 4 week old NOD mice with their ANOVA p-values (Benjamini-Hochberg, p<0.05) and fold-changes relative to two control strains, NON and C57BL/6.** A. 298 probesets of significantly lower expression in NOD mice; and B. 29 probesets of significantly higher expression in NOD mice.
(XLS)
