# Supplementary figures and images for: Molecular Phenotyping of Immune Cells from Young NOD Mice Reveals Abnormal Metabolic Pathways in the Early Induction Phase of Autoimmune Diabetes
Source: PLoS One. 2012 Oct 11;7(10):e46941. doi: 10.1371/journal.pone.0046941 (PMC3469658; doi:10.1371/journal.pone.0046941)

## Slide 1
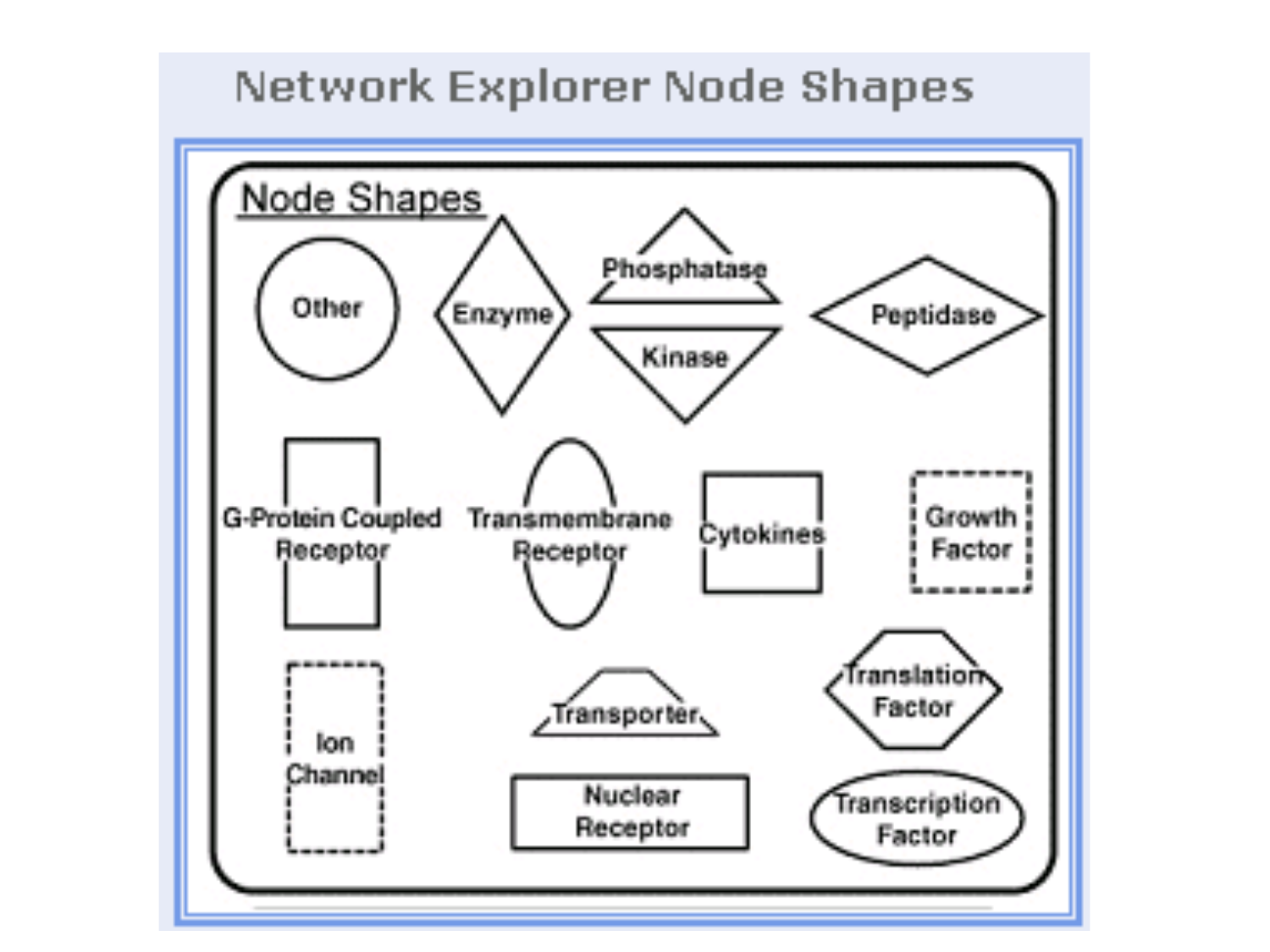

Supplement: Figure S1 — Symbols in Ingenuity Pathway Analysis networks and their meaning. (PPT) [file pone.0046941.s001.ppt]

## Slide 1
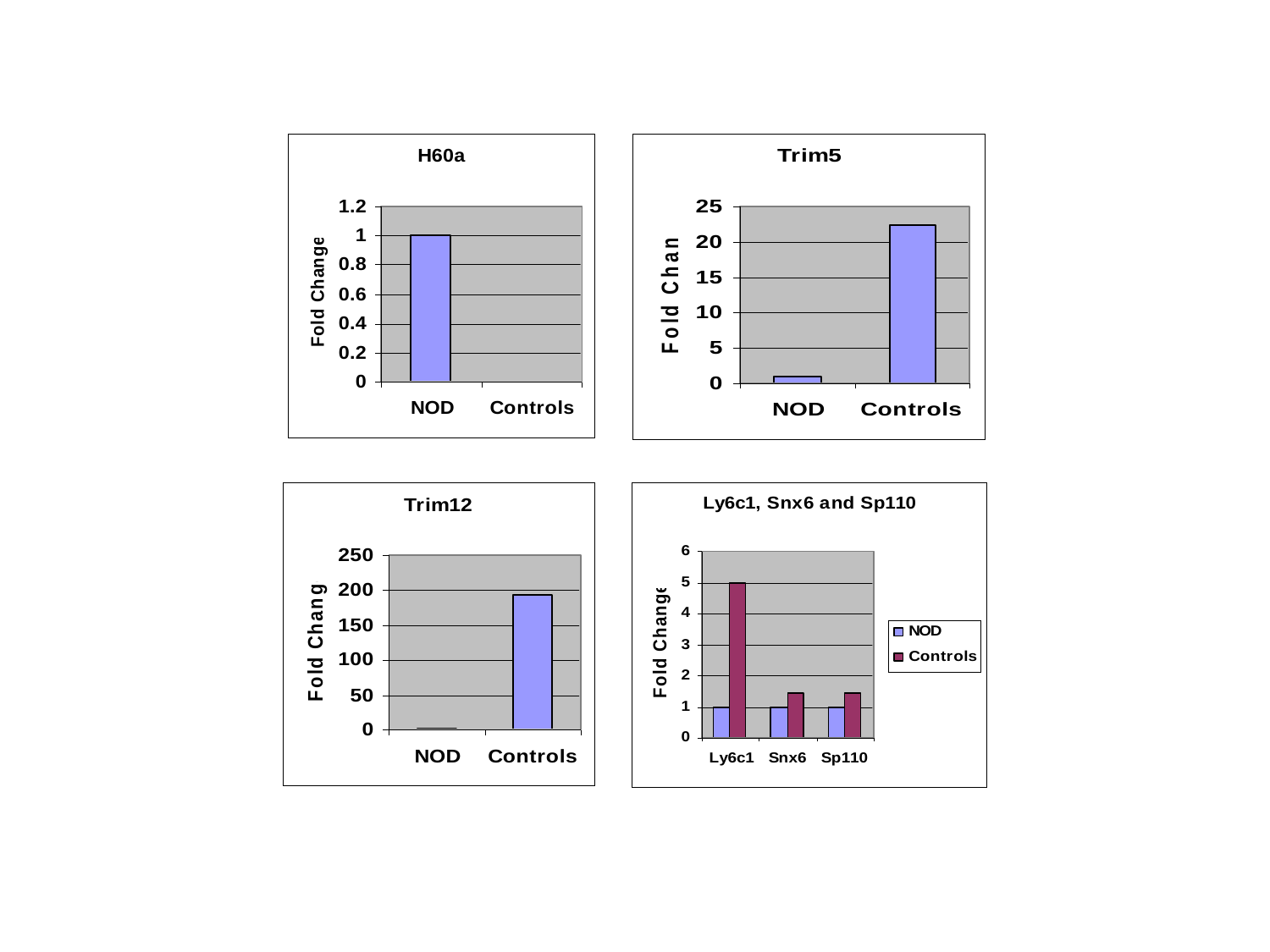

Supplement: Figure S2 — Validation of microarray data by quantitative real-time PCR. Quantitative real-time PCR was performed for H60a, Trim5, Trim12, Ly6c1, Snx6 and Sp110. Fold change values represent expression levels of genes in control strains (NON and C57BL/6 mice) relative to NOD mice. The results are consistent with the microarray data results. (PPT) [file pone.0046941.s002.ppt]

## Slide 1
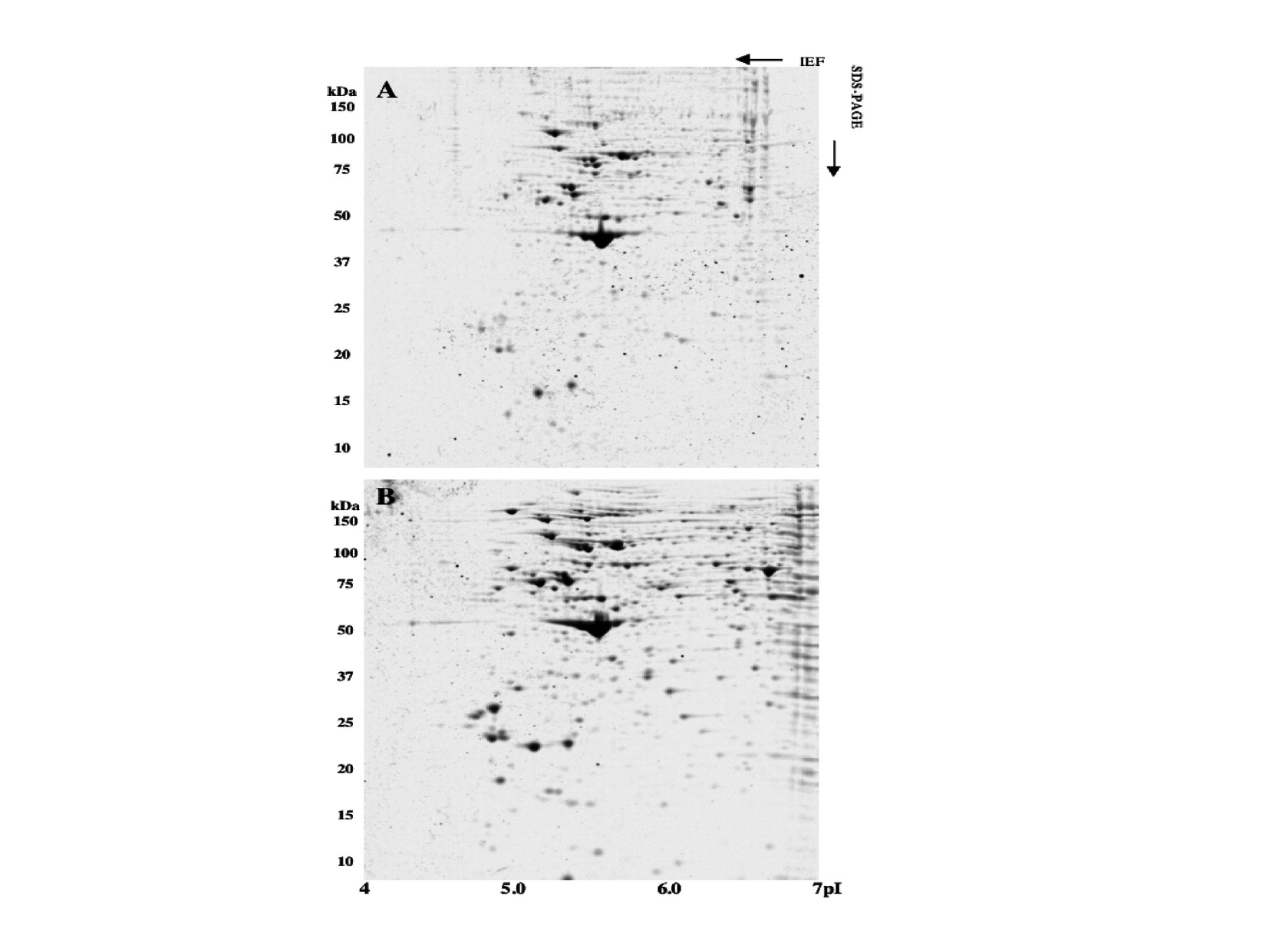

Supplement: Figure S3 — Sample 2D gel images of protein extracts from spleen leukocytes of one control strain (C57BL/6, A) and NOD mice (B). The approximate pI and molecular mass in kDa is given on the x and y axes, respectively. (PPT) [file pone.0046941.s003.ppt]

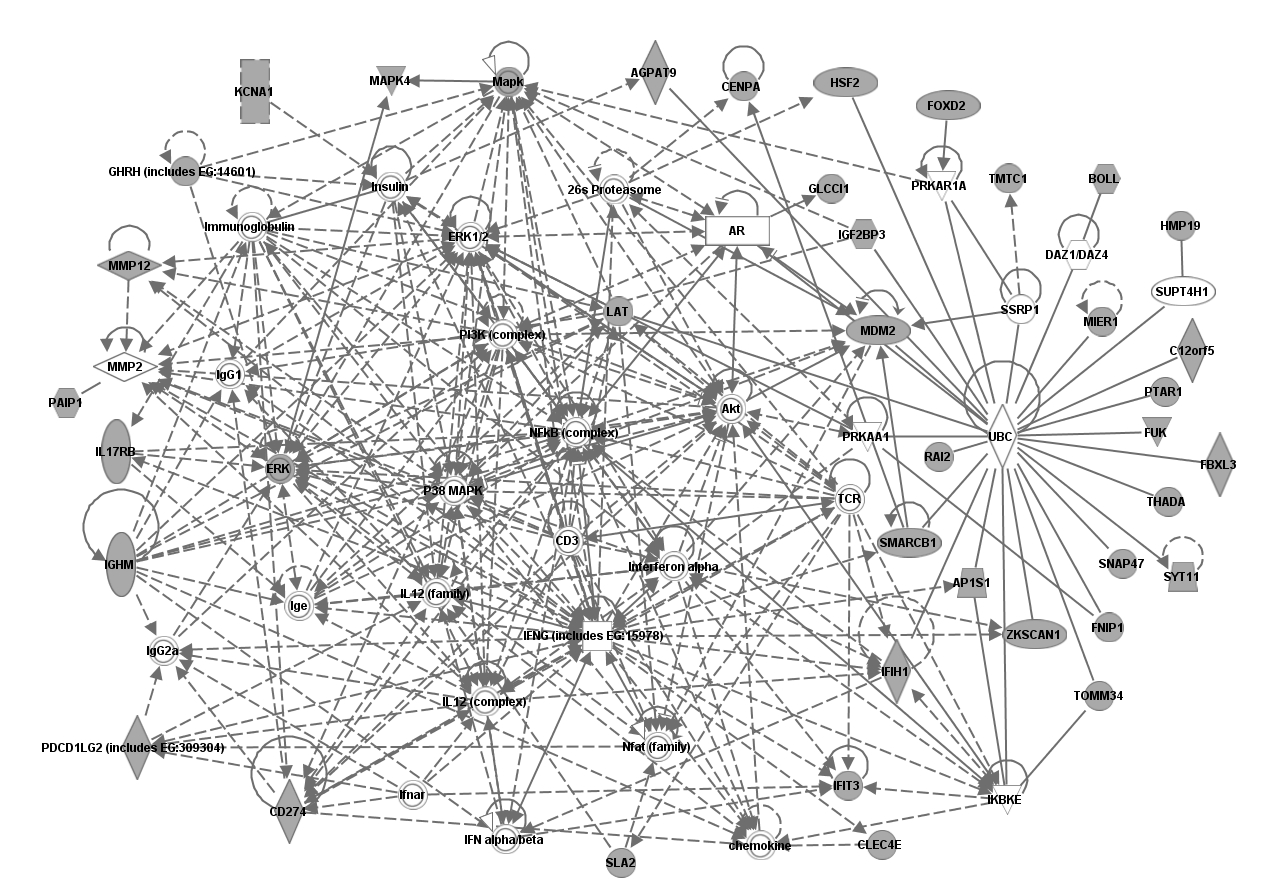

Supplement: Figure S4 — Transcriptome network created by Ingenuity Pathway Analysis from genes uniquely differentially expressed in NOD mice between 2 weeks and 4 weeks of age. The merged network was generated from the list of transcripts uniquely differentially expressed in spleen leukocytes of NOD mice between 2 weeks and 4 weeks of age in comparison to two control strains, NON and C57BL/6. It represents the three topmost major networks. The genes derived from our uploaded gene list (focus genes) are represented by gray icons. White icons represent genes (or endogenous chemicals) derived solely from the IPA knowledge base and that could be algorithmically connected to the focus genes. (TIFF) [file pone.0046941.s004.tif]

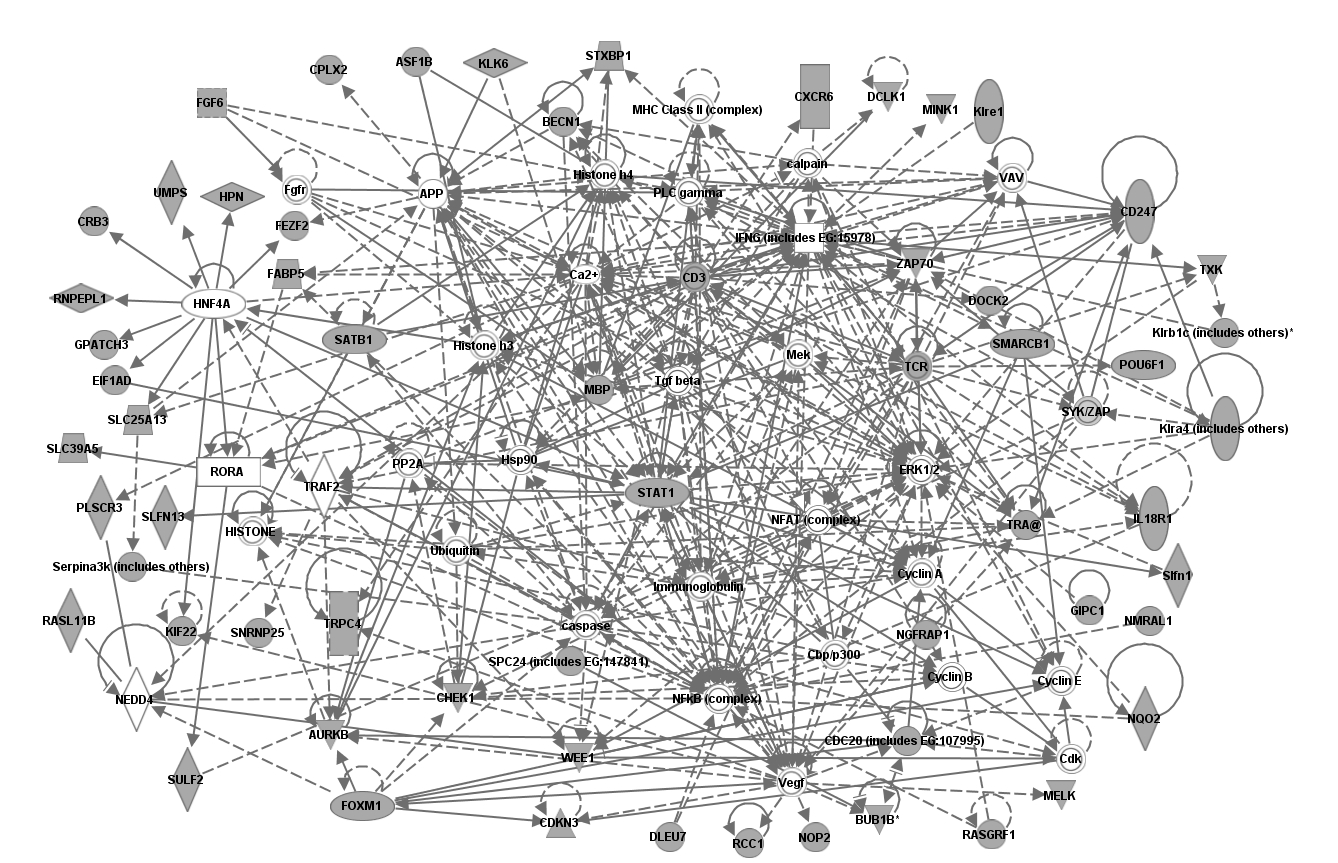

Supplement: Figure S5 — Transcriptome network created by Ingenuity Pathway Analysis from genes uniquely differentially expressed in NON mice between 2 weeks and 4 weeks of age. The merged network was generated from the list of transcripts uniquely differentially expressed in spleen leukocytes of NON mice between 2 weeks and 4 weeks of age in comparison to NOD and C57BL/6. It represents the three topmost major networks. The genes derived from our uploaded gene list (focus genes) are represented by gray icons. White icons represent genes (or endogenous chemicals) derived solely from the IPA knowledge base and that could be algorithmically connected to the focus genes. (TIFF) [file pone.0046941.s005.tif]

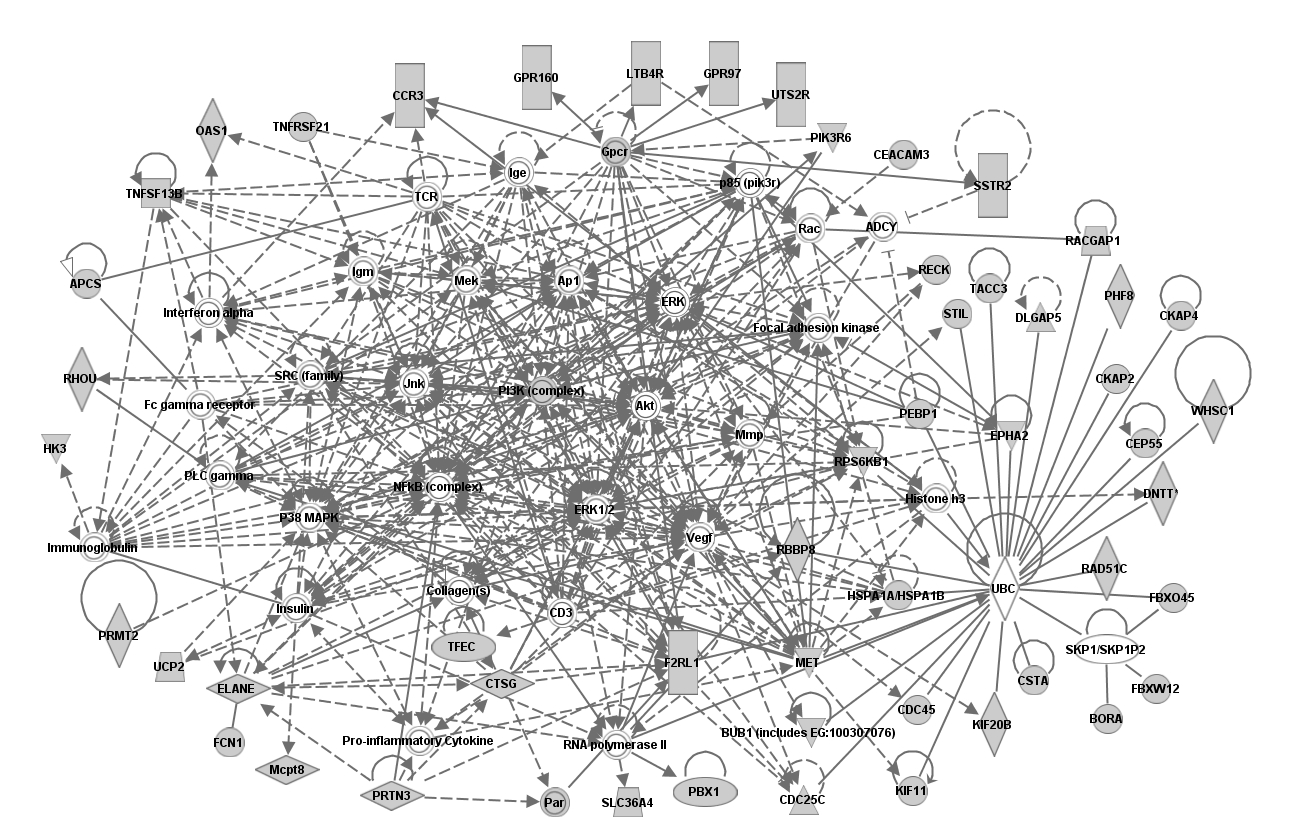

Supplement: Figure S6 — Transcriptome network created by Ingenuity Pathway Analysis from genes uniquely differentially expressed in C57BL/6 mice between 2 weeks and 4 weeks of age. The merged network was generated from the list of transcripts uniquely differentially expressed in spleen leukocytes of C57BL/6 mice between 2 weeks and 4 weeks of age in comparison to NOD and NON mice. It represents the three topmost major networks. The genes derived from our uploaded gene list (focus genes) are represented by gray icons. White icons represent genes (or endogenous chemicals) derived solely from the IPA knowledge base and that could be algorithmically connected to the focus genes. (TIFF) [file pone.0046941.s006.tif]
