# Supplementary material for: Molecular Phenotyping of Immune Cells from Young NOD Mice Reveals Abnormal Metabolic Pathways in the Early Induction Phase of Autoimmune Diabetes
Source: PLoS One. 2012 Oct 11;7(10):e46941. doi: 10.1371/journal.pone.0046941 (PMC3469658; doi:10.1371/journal.pone.0046941)
Supplement: Table S1 — Genes and primers/probes used for quantitative Real-time PCR. (DOC) [file pone.0046941.s007.doc]

**Table S1. List of genes and primers/probes used for quantitative Real-time PCR.**

| **Transcript** | **Accession No.** | **UPL Probe No.** | **Primer sequence** |
| --- | --- | --- | --- |
| H60a | NM_010400.2 | 17 | Left ATGCAGGTCTCCCCTAGCTT  Right TCACACAGACTCAATGCAGGT |
| Trim5 | XM_992714.3 | 68 | Left GGCCACAAAACAGCTCTCAT  Right TCAGCCTTTGCAGAACTACCT |
| Trim12 | AF220126.1 | 68 | Left AGCACCGTGGTCACAAAAC  Right CCATCAGCCTTTGCAGAACT |
| Ly6c1 | NM_010741.3 | 98 | Left GTCCACATCTGACAGAACTTGC  Right TCCATCCTCAGAGAAGGGTTC |
| Snx6 | NM_026998.3 | 17 | Left CTAGAACTGAAGCATGCAAAGG  Right CTCCATTTAAAACCGCAAGG |
| Sp110 | NM_175397.4 | 93 | Left CCGGGACAATTCCTTCATC  Right ATTGTGCACCACTTTGGACA |

UPL ~ Universal Probe Library (Roche)
